# Thickness-directed oxide-templated conversion of sputtered tungsten films into WS_2_ nanotubes

**DOI:** 10.1039/d6tc02147f

**Published:** 2026-07-30

**Authors:** C. Soukaras, J. I. Echavarria, D. J. Degeling, J. Hernandez-Rueda, S. Conesa-Boj

**Affiliations:** a Kavli Institute of Nanoscience, Delft University of Technology Delft 2628 CJ The Netherlands s.conesaboj@tudelft.n; b Optics Department, Faculty of Physics, University Complutense of Madrid. Plaza de Ciencias 1 28040 Madrid Spain; c Instituto de Química Física Blas Cabrera, IQF-CSIC Serrano 119 28006 Madrid Spain

## Abstract

Controlling the topology of curved transition-metal dichalcogenides remains challenging because shell formation, precursor conversion and hollowing occur simultaneously during growth. Here, we show that the thickness of sputtered tungsten (W) films provides a direct handle for selecting WS_2_ nanotube topology during charcoal-assisted oxide-template sulfurization. Varying the initial W thickness from 1 to 100 nm drives a transition from laterally extended WS_2_-like domains to hollow nanotubes and, finally, to retained-core nanotubes exhibiting shell-front mismatch and spiral-like shell wrapping. Sulfur-free controls, Raman spectroscopy, grazing-incidence X-ray diffraction, transmission electron microscopy and elemental mapping reveal that this transition is governed by the continuity, accessibility, and recession of WO_*x*_-derived precursor structures. As an initial test of functionality, multiphoton microscopy further shows that the oxide-core nanotube assemblies are optically active nonlinear emitters, generating second-harmonic, sum-frequency and four-wave-mixing signals that are readily detected under conditions where a WS_2_ monolayer reference is near the detection limit. These results establish film thickness as a synthetic handle for curved transition-metal dichalcogenide architectures and identify these self-formed nanostructures as a promising platform for nonlinear nanophotonics.

## Abstract 2

W film thickness alone selects the topology of oxide-templated WS_2_: hollow at 5 nm, a WO_*x*_ remnant at 10 nm, and a retained core at 100 nm, turning a growth by-product into a design parameter.

## Graphical abstract

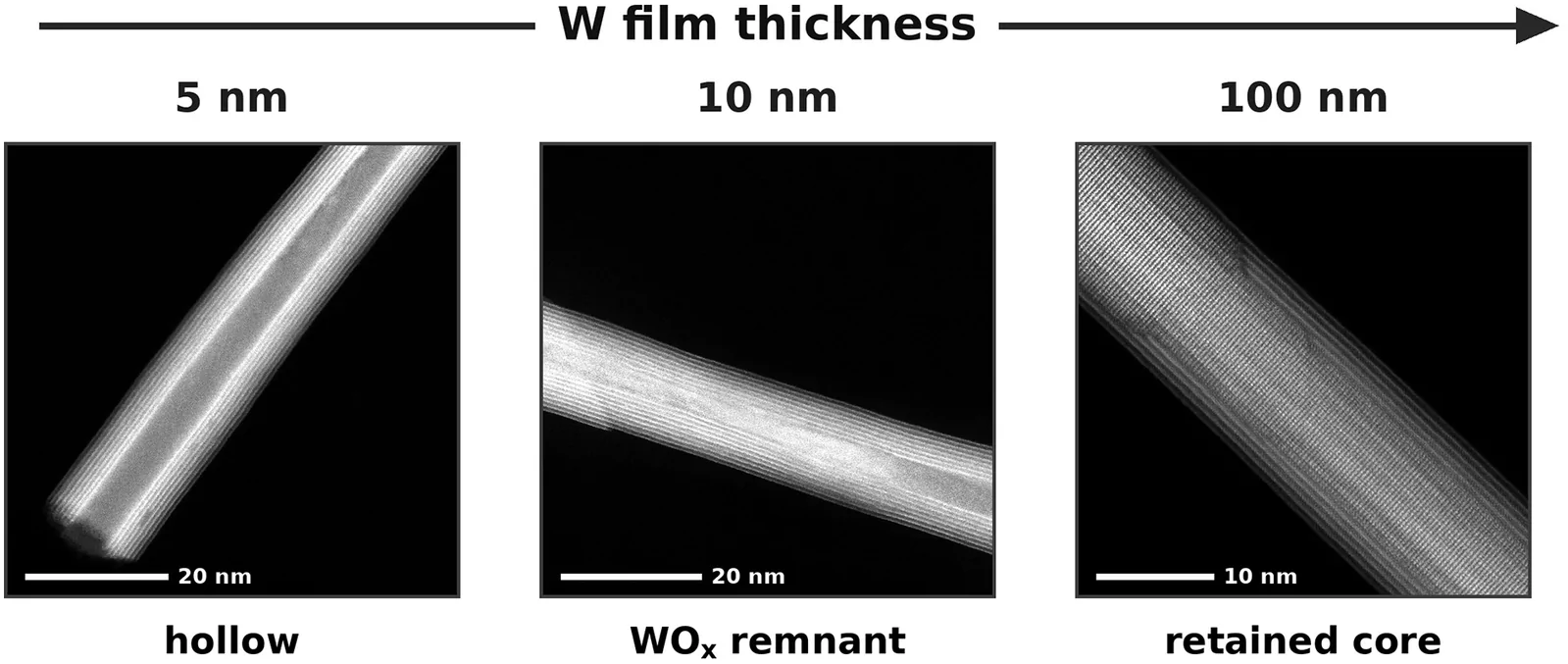

## 1. Introduction

Curved transition-metal dichalcogenides (TMDs) provide a platform in which the geometry of a layered crystal can be used to modify its electronic, optical and structural response. WS_2_ nanotubes are a prototypical example: they are curved, multiwalled van der Waals crystals in which layered WS_2_ is closed into a one-dimensional morphology. Since their first observation as concentric polyhedral and cylindrical structures formed under sulfurizing conditions,^1^ these nanotubes have provided a model system for studying how topology modifies the behavior of layered materials. Their properties depend on structural parameters such as diameter, wall number, curvature and chirality, which can influence electronic, optical and structural response.^2–5^ Unlike planar WS_2_, however, nanotube topology is established during growth, when shell formation, hollowing and defect generation occur simultaneously with chemical conversion of the tungsten-containing precursor. Controlling WS_2_ nanotube synthesis therefore requires understanding not only the final crystal structure, but also how precursor morphology evolves during sulfurization.

Most mechanistic understanding of WS_2_ nanotube formation has come from oxide-template routes. In these routes, WO_*x*_, WO_3−*x*_ or W_18_O_49_ nanowires are converted into WS_2_ nanotubes while largely preserving the anisotropic precursor geometry.^6–9^ This process is commonly described as a surface-inward reaction, in which sulfur-containing species react at the oxide surface and the conversion front advances towards the core. Recent imaging studies have refined this picture by showing that the first WS_2_ shells can restrict further sulfur transport, while anisotropic volatilization and recession of the tungsten suboxide core contribute to hollowing and internal shell formation.^10,11^ These results show that the oxide precursor is not only a geometric template, but also an active chemical intermediate that determines sulfur access, core recession and the final nanotube architecture.

A complementary route starts from metallic W thin films rather than preformed oxide nanowires. In this case, the initially planar film evolves during heating, oxidation, reduction, and sulfurization before nanotube formation can occur. Previous work has linked WS_2_ nanotube growth from W films to precursor-film thickness, stress accumulation and curling or wrapping of initially formed WS_2_ layers.^12,13^ Related studies on sulfurized transition-metal seed layers and W films have shown that the starting metal thickness can influence whether growth yields vertical, horizontal, tubular or laterally extended TMD morphologies.^14,15^ Yet the connection between thin-film sulfurization and oxide-template conversion remains unresolved. In particular, it is unclear how the thickness of a sputtered W film controls the formation, continuity and accessibility of WO_*x*_-derived precursor structures, or how these precursor changes select between dense nanotube growth, island-associated nanotube nucleation and lateral WS_2_ crystallization.

The structural diversity produced by oxide-template conversion is also relevant for nonlinear optics. Monolayer WS_2_ already exhibits an unusually strong nonlinear optical response, while strain, broken-symmetry spiral morphologies and nanotube curvature have all been shown to modify nonlinear emission in TMD systems.^16–20^ In individual WS_2_ nanotubes, for example, second-harmonic generation has revealed chiral domains and excitonic states, highlighting the sensitivity of nonlinear emission to curvature, symmetry and local crystal structure.^19^ The oxide-core nanotubes produced by incomplete oxide-template conversion add further structural ingredients, including finite wall number, axial heterogeneity, shell-front mismatch, local shell wrapping and an oxide-derived internal core, that could likewise influence their optical response. This motivates an initial test of whether these self-formed curved structures are optically active, beyond their interest as a model system for oxide-template conversion.

Here we show that the initial thickness of sputtered W films directs WS_2_ nanotube formation by controlling the continuity, accessibility and recession of WO_*x*_-derived precursors formed during charcoal-assisted chemical vapor deposition (CVD) sulfurization. By comparing W films with nominal thicknesses of 100, 10, 5 and 1 nm processed in the same face-down charcoal-assisted CVD geometry, we identify a thickness-selected transition from dense nanotube-rich morphologies to island-associated nanotube nucleation and, finally, predominantly laterally extended WS_2_-like domains. Electron microscopy, elemental mapping, Raman spectroscopy and grazing-incidence X-ray diffraction (GIXRD) support a mechanism in which film thickness regulates precursor continuity, sulfur-access geometry and oxide-core recession. The 5 nm films form discontinuous WO_*x*_-derived domains that seed sparse hollow nanotubes, whereas 10 nm films produce axially heterogeneous nanotubes with local WO_*x*_-derived remnants. In the 100 nm regime, incomplete conversion produces axially heterogeneous nanotubes that retain WO_*x*_-derived cores over extended segments, with nonuniform shell fronts and local shell wrapping. After establishing this thickness-directed conversion sequence, we use nondegenerate multiphoton microscopy as a first probe of optical functionality, focusing on the oxide-core nanotubes from the 100 nm growth regime. These measurements show that the nanotube assemblies are efficient nonlinear emitters, producing localized second-harmonic generation (SHG), sum-frequency generation (SFG) and four-wave mixing (FWM) that are clearly detected where a monolayer reference is near the detection limit, and whose FWM resonance is spectrally shifted relative to the monolayer. Together, these results bridge thin-film sulfurization and oxide-template conversion, identify W film thickness as a direct handle for selecting WS_2_ nanotube topology, and point to these self-formed curved heterostructures as functional building blocks for nonlinear nanophotonics.

## 2. Results and discussion

### 2.1. Thickness-directed oxide-templated conversion under charcoal-assisted sulfurization

To determine how the initial W film thickness affects WS_2_ nanotube formation, sputtered W films on Si with nominal thicknesses of 100, 10, 5, and 1 nm were processed by charcoal-assisted CVD sulfurization. All samples in the thickness series were treated in the same face-down geometry, in which the W-coated substrate was placed above charcoal powder while sulfur vapor was supplied from the upstream furnace zone. The substrate temperature, growth time, and sulfur/charcoal loadings were comparable across the series, with sample-specific growth conditions summarized in Table S1. The final Ar flow was 110 sccm for the 10, 5 and 1 nm films and 150 sccm for the representative 100 nm film; an additional 100 nm sample processed at 110 sccm also produced a nanotube-rich morphology (Fig. S4a–d). The charcoal-assisted geometry promotes the formation of WO_*x*_-derived precursor structures, which are subsequently converted into WS_2_ nanostructures during sulfurization. Control experiments on W foil show that charcoal powder placed beneath the face-down substrate produces denser, longer, and more spatially uniform WO_*x*_ nanowires than brief carbon-tape contact, with the strongest effect observed in the region directly above the charcoal mount (Fig. S1–S3). These controls establish a precursor-forming environment in which changes in final WS_2_ morphology can be compared as a function of starting W film thickness.

**Fig. 1:**
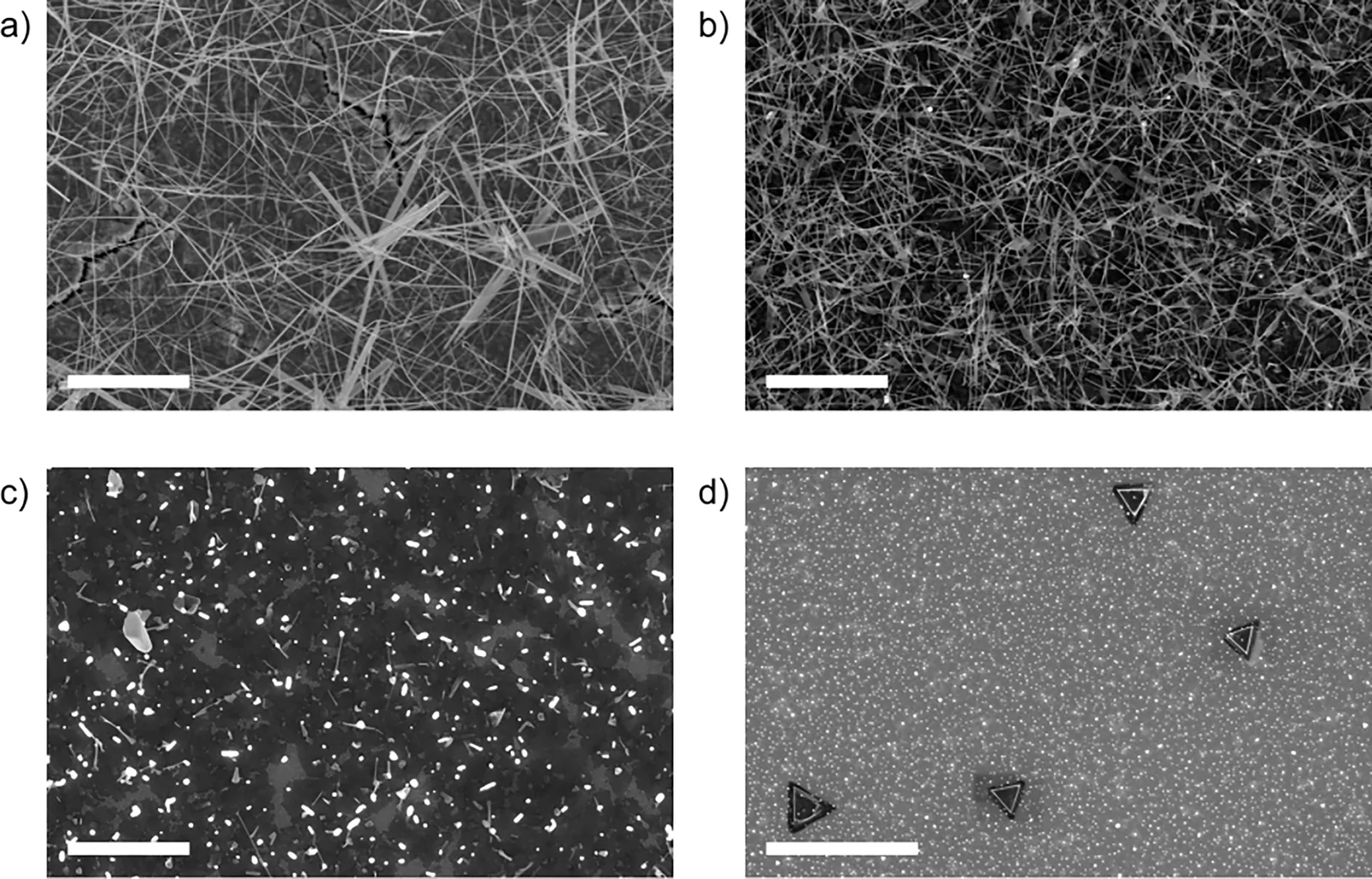
Thickness-dependent morphology of WS_2_ nanostructures grown from sputtered W films. SEM images of samples prepared from initial W film thicknesses of (a) 100 nm, (b) 10 nm, (c) 5 nm, and (d) 1 nm under the charcoal-assisted sulfurization conditions summarized in Table S1. The 100 nm film yields a dense network of elongated WS_2_ nanotube-like structures with localized delamination, whereas the 10 nm film forms a dense nanotube network without obvious delamination. The 5 nm film produces a sparser population of shorter nanotubes together with bright island-like domains and darker laterally grown WS_2_ regions. The 1 nm film suppresses tubular growth and instead yields predominantly laterally extended WS_2_-like domains with residual bright island-like features. Scale bars: (a–c) 2 µm and (d) 10 µm.

Scanning electron microscopy (SEM) reveals a pronounced dependence of the final morphology on the initial W thickness (Fig. 1). The 100 nm W film produces a dense network of elongated WS_2_ nanotube-like structures across the substrate (Fig. 1a). Local delamination is also observed, consistent with stress accumulation during conversion of the thicker W-derived layer. Reducing the W thickness to 10 nm preserves dense nanotube formation, but without obvious delamination in the representative region shown (Fig. 1b). These two thicknesses therefore provide sufficient W-derived precursor continuity to support high-density nanotube growth.

Further reducing the W thickness changes the growth pathway. The 5 nm W film yields a sparser population of shorter nanotubes, together with bright island-like domains and darker regions attributed to laterally grown WS_2_ (Fig. 1c). At 1 nm, tubular growth is largely suppressed, and the surface is instead dominated by laterally extended WS_2_-like domains with residual bright island-like features (Fig. 1d). Additional SEM images acquired from spatially distinct regions of each substrate show that this thickness-dependent morphology is reproducible across the sampled areas (Fig. S4). Nanotube length and diameter statistics measured after transfer to transmission electron microscopy (TEM) grids further support this thickness-dependent morphology (Fig. S17). The 100 nm-derived nanotubes show the largest mean diameter, 48.58 ± 15.58 nm, and the broadest diameter distribution, together with a mean length of 1.52 ± 0.73 µm. Nanotubes derived from 10 and 5 nm W films have smaller, broadly comparable mean diameters of 26.85 ± 8.37 nm and 28.51 ± 8.58 nm, respectively, and shorter mean lengths of 0.623 ± 0.211 µm and 0.514 ± 0.251 µm. Because these measurements were obtained from transferred TEM-grid samples with *N* = 28 nanotubes per thickness, the values are used to support relative morphology trends rather than to quantify absolute substrate-scale growth yields.

This progression defines a transition from continuous nanotube-rich growth to island-associated nanotube nucleation and finally to predominantly lateral WS_2_-like domains. The trend indicates that the initial W film thickness does not simply determine the amount of available tungsten. Instead, it regulates the continuity, anisotropy and sulfur accessibility of the WO_*x*_-derived precursor formed during charcoal-assisted CVD, thereby selecting the subsequent sulfurization pathway. This behavior differs from the vertical-to-horizontal growth transition reported for sulfurized transition-metal seed layers, where the main outcome is a change in layered-film orientation rather than selection of a nanotube-growth pathway.^14^ It is more closely related to oxide-template routes, in which WS_2_ nanotube formation is favored when sulfurization proceeds through an anisotropic, reactive WO_*x*_-derived precursor that retains a one-dimensional geometry during conversion.^8,10^ In the present geometry, charcoal-assisted CVD provides a precursor-forming environment, as supported by the W-foil controls and by GIXRD/Raman evidence for tungsten oxide/suboxide formation (Fig. S1–S3 and S6). Taken together, the thickness series in Fig. 1 indicates that WO_*x*_-derived precursor formation alone is not sufficient to determine the final morphology. Rather, nanotube-rich growth is favored when the oxide-derived precursor remains sufficiently continuous and accessible during sulfurization, whereas discontinuous or ultrathin precursors lead to island-associated nanotube nucleation or predominantly laterally extended WS_2_-like domains.

### 2.2. Sulfur-free annealing reveals discontinuous WO_*x*_-derived precursors

To test whether the thickness-dependent morphologies in Fig. 1 arise from differences in oxide-precursor formation, we isolated the precursor stage in the thinnest nanotube-forming regime. A 5 nm W/Si sample was processed in the same face-down charcoal-assisted CVD geometry used for nanotube growth, but without sulfur. This sulfur-free control separates WO_*x*_-derived precursor formation from subsequent WS_2_ conversion.

**Fig. 2:**
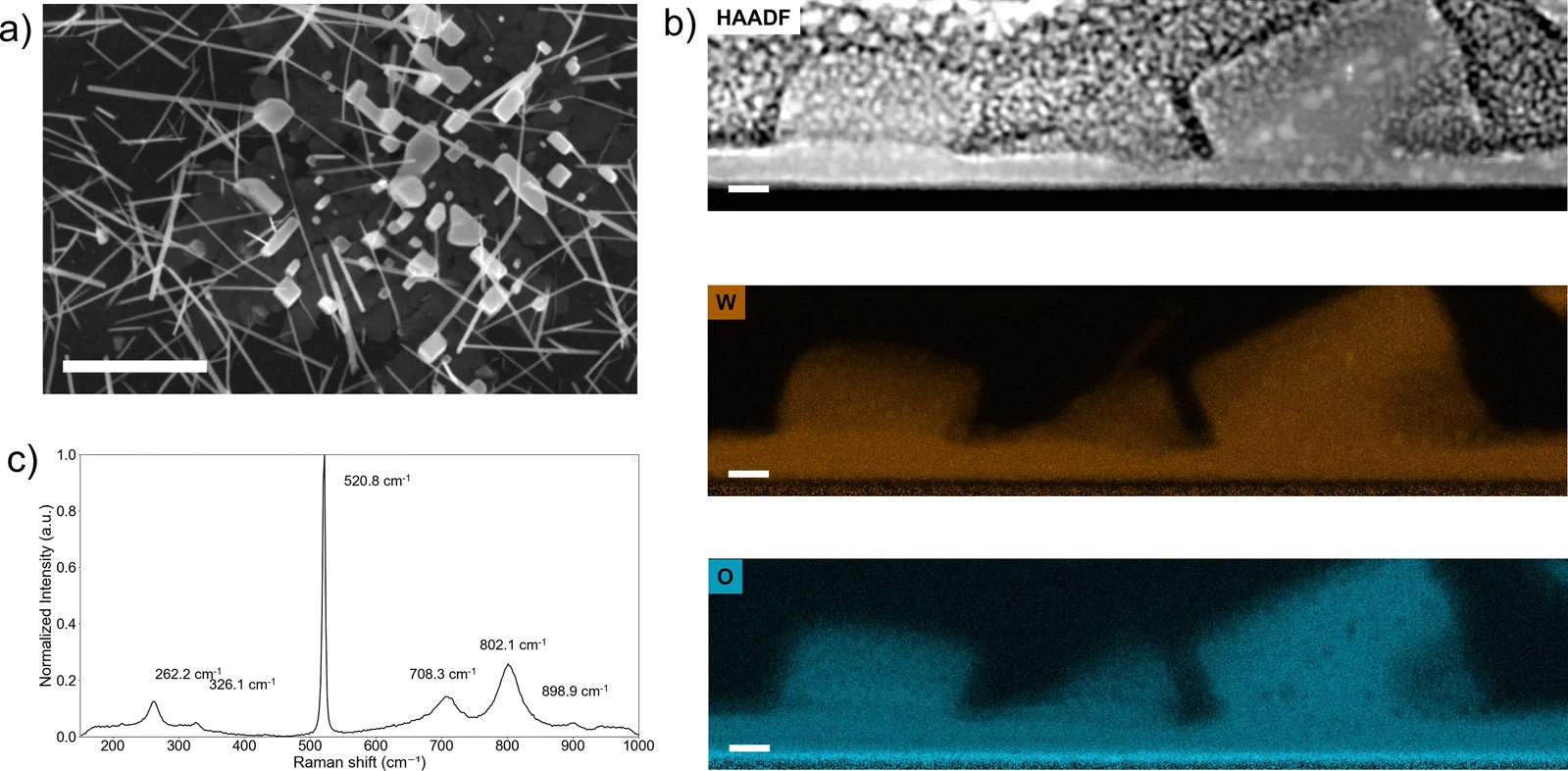
Sulfur-free precursor evolution in the 5 nm sputtered-W regime. (a) SEM image of the sulfur-free processed 5 nm W/Si sample, showing nanowire-like structures, island-like domains and flatter surrounding regions. (b) Cross-sectional ADF-STEM image and corresponding STEM-EDX W and O maps of an island-containing lamella, showing W/O-rich precursor domains. (c) Raman spectrum of the sulfur-free processed film, showing tungsten oxide/suboxide vibrational features together with Si substrate features. Scale bars: (a) 0.5 µm; (b) 20 nm.

Before annealing, the sputtered 5 nm W film appears comparatively smooth and laterally continuous by SEM (Fig. S5a). After sulfur-free charcoal-assisted annealing, the film reorganizes into a heterogeneous surface containing nanowire-like structures, island-like domains and flatter surrounding regions (Fig. 2a and Fig. S5b). Cross-sectional annular dark-field scanning transmission electron microscopy (ADF-STEM) imaging and STEM energy-dispersive X-ray spectroscopy (STEM-EDX) mapping of a focused-ion-beam-prepared lamella confirm W and O enrichment in these island-like domains (Fig. 2b). Raman spectroscopy of the sulfur-free processed film shows bands at 262.2 and 326.1 cm^−1^, together with higher-frequency bands at 708.3 and 802.1 cm^−1^ (Fig. 2c). These features are consistent with reported O–W–O bending/deformation and stretching modes of tungsten oxide and substoichiometric tungsten oxide phases.^21–24^ A weaker feature near 898.9 cm^−1^ is consistent with a W=O stretching contribution reported for reduced tungsten oxides,^23,25^ while the intense peak at 520.8 cm^−1^ and the broad feature near 970 cm^−1^ arise from the Si substrate. Additional precursor characterization, including SEM, GIXRD, Raman analysis, high-resolution TEM (HRTEM) and extended elemental mapping, are provided in Fig. S5–S8 and Tables S3–S5.

These results show that the nominally continuous 5 nm W film breaks up into discontinuous WO_*x*_-derived domains before sulfurization. This precursor morphology provides a structural basis for the sparse, island-associated nanotube growth observed after full sulfurization of 5 nm W films. Rather than acting as a continuous tungsten reservoir, the 5 nm film forms localized W/O-rich domains that can seed nanotube nucleation while surrounding regions favor laterally extended WS_2_-like material.

### 2.3. Island-mediated nucleation and hollow-shell formation in the 5 nm regime

**Fig. 3:**
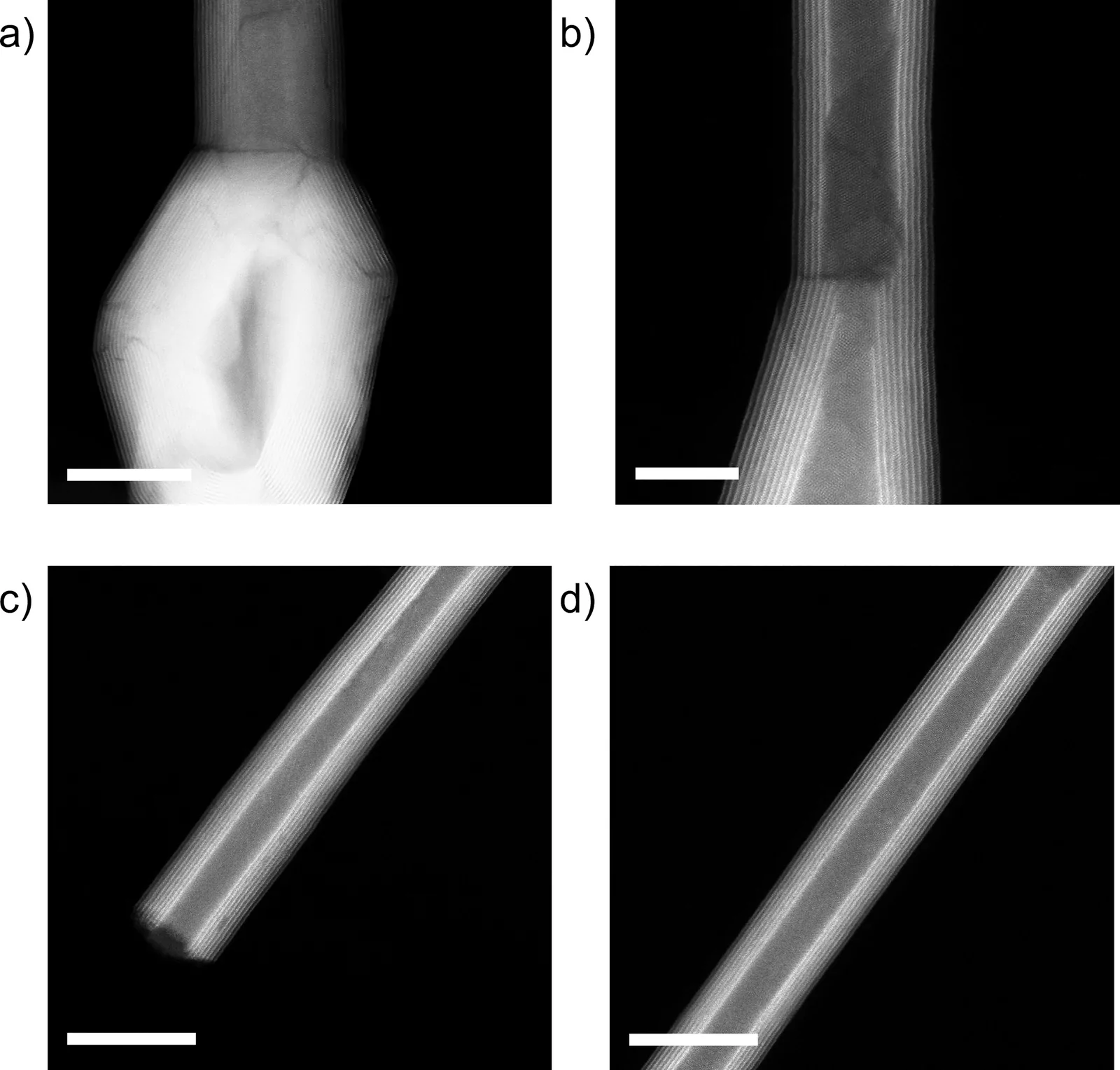
Island-mediated nucleation and hollow-shell formation in WS_2_ nanotubes grown from 5 nm W films. (a) Early-stage HAADF-STEM image acquired after 1 s of sulfurization, showing a nanotube-like segment attached to an island-like root region. (b) HAADF-STEM image after 30 min of sulfurization, showing a more developed island-associated nanotube base with layered WS_2_ walls emerging from the broader precursor-derived region. (c) HAADF-STEM image of a representative 30 min-grown nanotube segment with an open hollow end and no obvious retained dense oxide-derived core. (d) High-resolution STEM image of a 30 min-grown nanotube body, showing concentric WS_2_ shells. Scale bars: (a) 20 nm, (b) 10 nm, and (c) and (d) 20 nm.

We next examined the 5 nm samples after short and extended sulfurization to connect the discontinuous precursor state with the resulting nanotube shell structure. After 1 s of sulfurization, high-angle annular dark-field STEM (HAADF-STEM) reveals a nanotube-like segment attached to a broader island-like root region (Fig. 3a). This early-stage morphology is consistent with nanotube nucleation from a localized oxide-derived precursor reservoir. After extended sulfurization for 30 min, the island-associated root region develops into a more defined nanotube structure, with layered WS_2_ walls emerging from the broader base (Fig. 3b). These representative early- and extended-growth snapshots support an island-mediated nucleation pathway in the 5 nm regime.

The 30 min-grown nanotubes frequently exhibit hollow interiors and concentric multilayer walls. A representative nanotube segment shows an open hollow end with no obvious retained dense oxide-derived core in HAADF-STEM contrast (Fig. 3c). High-resolution imaging further shows regular concentric WS_2_ shells along the nanotube body (Fig. 3d). Additional high-resolution STEM (HRSTEM) images in Fig. S9 and S10 show that this concentric shell morphology can persist along substantial portions of the nanotube length, including open-ended segments and regions with approximately symmetric shell numbers on opposing walls. The same supplementary images also show occasional local shell interruptions and recovery of shell symmetry, indicating that defects occur locally but do not dominate the 5 nm growth regime.

Together, these observations show that the 5 nm film follows an island-mediated conversion pathway. Localized WO_*x*_-derived domains initiate nanotube growth, while subsequent sulfurization and oxide-core recession produce short hollow WS_2_ nanotubes with comparatively regular concentric shells. In this oxide-templated route, the effective growth rate is set by the sulfurization kinetics rather than by tungsten supply: nanotube nucleation occurs within seconds on accessible precursor surfaces, while subsequent conversion is limited by sulfur transport through the already-formed WS_2_ shells and by oxide-core recession, so that the final morphology reflects the balance between this conversion rate and the local precursor volume.

### 2.4. Axially heterogeneous conversion and local shell wrapping in the 10 nm regime

**Fig. 4:**
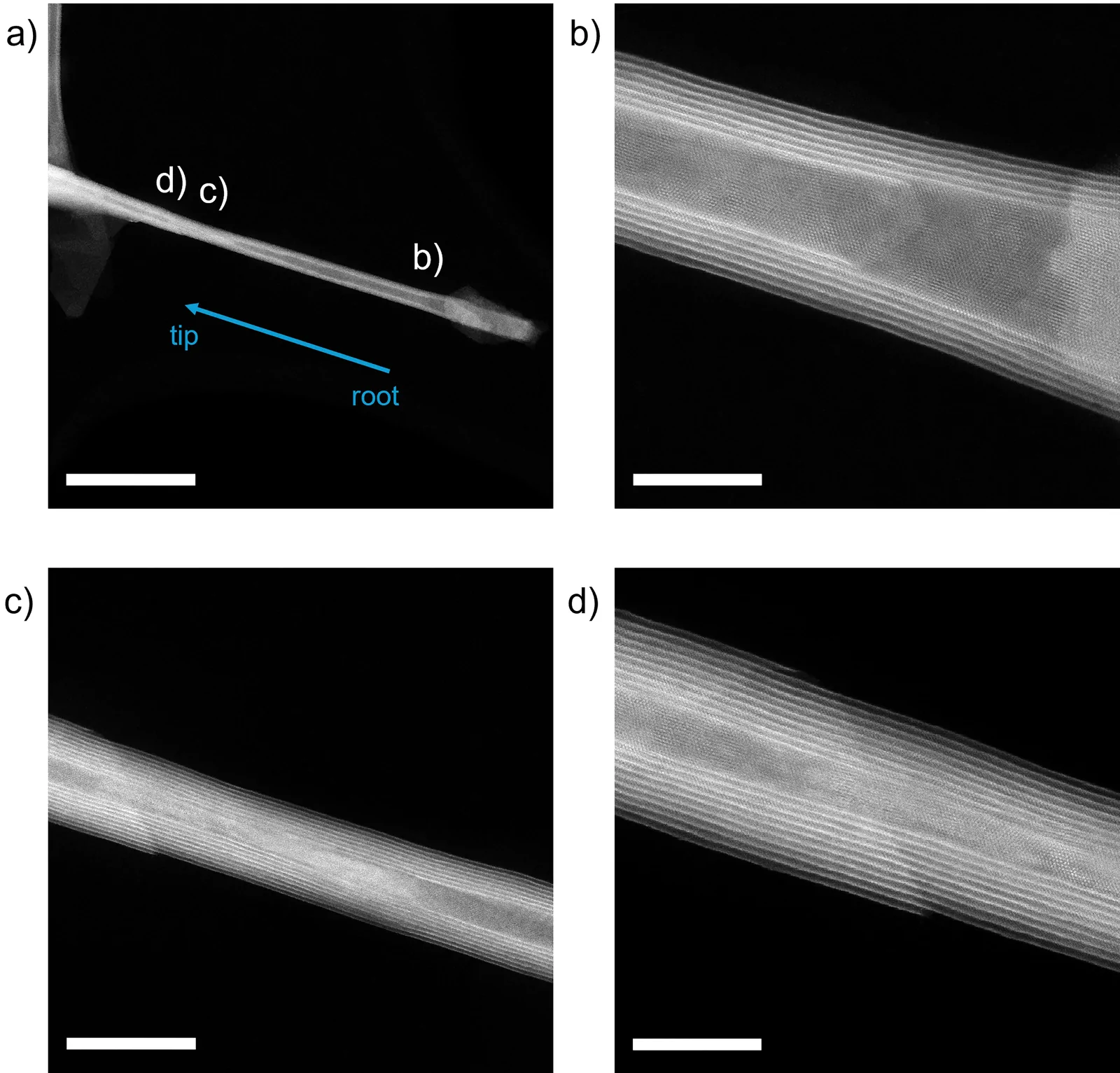
Axially heterogeneous conversion and local shell wrapping in WS_2_ nanotubes grown from 10 nm W films. (a) Low-magnification HAADF-STEM image of a representative nanotube grown from a 10 nm sputtered W film, showing the root-to-tip direction and the regions analyzed at higher magnification. (b) Higher-magnification image of a root-proximal region, where comparatively symmetric multilayer WS_2_ shells are established. The apparent projected diameter is approximately 15.8 nm. (c) Middle region of the same nanotube, showing local diameter narrowing to approximately 13.6 nm around a high-contrast axial remnant consistent with locally retained WO_*x*_-derived material. (d) Tip-proximal region of the same nanotube, showing re-expansion of the shell envelope to approximately 15.1 nm and spiral-like local shell wrapping. Scale bars: (a) 100 nm; (b) 10 nm; (c) 20 nm; (d) 10 nm.

Increasing the initial W thickness from 5 to 10 nm restores dense nanotube growth while preserving a precursor volume small enough for substantial conversion during sulfurization. HAADF-STEM imaging of a representative 10 nm-derived nanotube reveals an axially heterogeneous conversion pathway (Fig. 4a). In a root-proximal region, the WS_2_ walls are comparatively symmetric and composed of well-registered multilayer shells (Fig. 4b). This region has an apparent projected outer diameter of approximately 15.8 nm, with adjacent shell spacings of about 0.61 nm, consistent with layered WS_2_. The expanded, concentric shell geometry resembles the hollow nanotubes obtained in the 5 nm regime, indicating that ordered radial shell formation can also occur in 10 nm-derived nanotubes when the local precursor is sufficiently converted.

The conversion state changes along the same nanotube. In the middle region, the apparent diameter decreases to approximately 13.6 nm around a high-contrast axial remnant (Fig. 4c). The combination of enhanced HAADF contrast and local diameter narrowing suggests delayed conversion or incomplete recession of a dense WO_*x*_-derived remnant. The surrounding shells retain an interlayer spacing close to 0.61 nm, showing that the wall remains WS_2_-like despite the local axial remnant. Line-profile analysis of a second representative 10 nm-derived nanotube further confirms the hollow wall structure and local shell-number asymmetry (Fig. S11). The profiles show high-contrast WS_2_ wall fringes separated by a lower-intensity hollow core, while also revealing different apparent shell numbers on opposing tube walls. Local FFT analysis of the remnant-rich and transition regions reveals additional lattice periodicities distinct from the dominant WS_2_ shell spacing (Fig. S12 and Table S6). These periodicities are consistent with oxygen-deficient WO_3−*x*_ or tungsten suboxide motifs,^23,26,27^ but the overlap among tungsten oxide and suboxide lattice spacings prevents assignment of a unique oxide phase from FFT alone. We therefore refer to this feature as a WO_*x*_-derived remnant.

Toward the tip-proximal region, the shell envelope re-expands to approximately 15.1 nm and the WS_2_ layers develop a spiral-like wrapping geometry (Fig. 4d). This axial sequence suggests that local retention of a dense WO_*x*_-derived remnant can constrain shell expansion in the middle segment, whereas continued conversion and shell-edge propagation toward the tip are associated with asymmetric shell wrapping. Together, these observations identify the 10 nm film as an intermediate conversion regime. The precursor is sufficiently continuous to support dense nanotube formation, but sulfurization and oxide-core recession do not proceed uniformly along the nanotube axis.

This geometry is consistent with oxide-template conversion, in which WS_2_ shells form around a WO_*x*_-derived precursor while the oxide-derived core recedes during sulfurization.^11,28^ The observed local shell wrapping is conceptually related to crystal-growth models in which persistent surface steps sustain spiral-like growth,^29,30^ but the present images do not resolve an axial screw dislocation. Thus, the 10 nm morphology is best described as axially heterogeneous oxide-template conversion with local shell wrapping, rather than direct screw-dislocation-driven growth.

### 2.5. Core retention and shell-front mismatch in the 100 nm regime

**Fig. 5:**
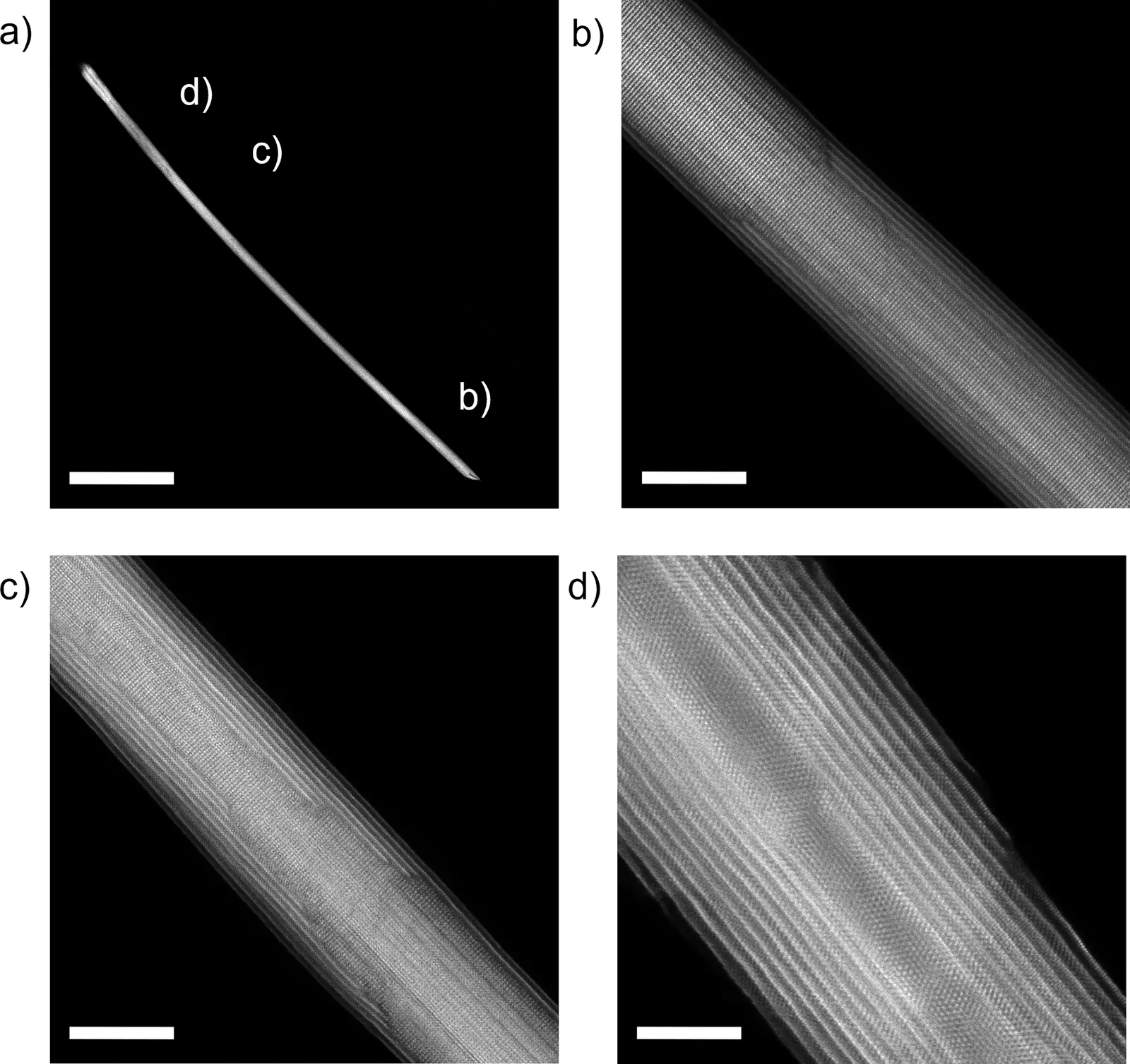
Core retention and shell-front mismatch in WS_2_ nanotubes grown from 100 nm W films. (a) Low-magnification HAADF-STEM image of a representative nanotube, showing the root-to-tip sequence of regions analyzed at higher magnification. (b) Root-proximal region: comparatively continuous WS_2_ shells surrounding a retained dense WO_*x*_-derived core. (c) Middle region: local shell-front mismatch, where WS_2_ layers at comparable radial positions become misregistered across an oblique discontinuity. (d) Tip-proximal region: spiral-like local shell wrapping of the WS_2_ layers. Scale bars: (a) 100 nm; (b) and (c) 10 nm; (d) 5 nm.

The 100 nm film is the largest initial W thickness examined in this series. Whereas the 5 nm regime produces hollow nanotubes from localized precursor reservoirs and the 10 nm regime shows localized axial remnant retention, the thicker precursor retains a dense core over extended nanotube segments. HAADF-STEM imaging of a representative 100 nm-derived nanotube shows multilayer WS_2_ shells surrounding a retained dense WO_*x*_-derived core (Fig. 5a and b). This retained-core morphology indicates that sulfurization and oxide-core recession are incomplete in the thick-film regime. Additional 100 nm-derived nanotubes with retained-core contrast and nonuniform shell development are shown in Fig. S13 and S14.

The shell structure varies along the nanotube axis, from root to tip. In the root-proximal region, WS_2_ layers remain comparatively continuous and well registered around the retained core (Fig. 5b). In the middle region, shells at comparable radial positions become locally misregistered, producing an oblique shell-front discontinuity across the tube wall (Fig. 5c). Toward the tip, the WS_2_ layers develop a spiral-like wrapping geometry (Fig. 5d), consistent with more advanced conversion and shell-edge propagation in this part of the tube.

These observations indicate that the 100 nm-derived nanotubes do not form through a single uniform inward-moving conversion front. Instead, WS_2_ shells form at accessible surfaces of the WO_*x*_-derived precursor while the dense core recedes or converts at different rates along the nanotube axis. Where shell fronts or locally growing shell segments meet out of registry, local step-like discontinuities can be generated. Related 100 nm-derived nanotubes show that such discontinuities can coincide with external or internal spiral-like WS_2_ layers (Fig. S15). Line-profile analysis across one mismatch region gives interlayer spacings close to 0.623 nm on both sides of the discontinuity, supporting local shell misregistration within WS_2_ rather than a gradual elastic distortion (Fig. S16).

This behavior is consistent with oxide-template conversion, in which WS_2_ shells form around a WO_*x*_-derived precursor while the oxide-derived core recedes during sulfurization.^10,11^ In the 100 nm regime, however, the precursor volume is sufficiently large that core recession remains incomplete and spatially nonuniform. The resulting shell-front mismatch can provide local step-like sites for secondary shell wrapping. This step-mediated interpretation is conceptually related to crystal-growth models in which persistent surface steps sustain spiral-like growth,^29,30^ but the present images do not require assignment to a resolved axial screw dislocation. Thus, the 100 nm film completes the thickness-dependent sequence: precursor continuity supports dense nanotube growth, but excessive precursor volume prevents uniform hollow conversion and promotes retained-core, shell-front-mismatch, and shell-wrapping morphologies.

### 2.6. WO_*x*_-derived retained-core WS_2_ nanotubes support nonlinear emission

The 100 nm growth regime produces a distinct retained-core WS_2_ nanotube architecture, in which multilayer WS_2_ shells surround a WO_*x*_-derived, tungsten-rich axial core and oxide-derived cores remain inside the nanotubes over extended segments. Because nonlinear emission in TMDs is broadly sensitive to curvature, dimensionality and local structure,^16,18,31^ we used these nanotubes to test whether the curved multiwall structures produced by this route are optically active, the present measurements probe this functionality rather than isolating the contribution of any individual structural feature. To this end, nanotubes from the 100 nm growth regime were dispersed in isopropanol, drop-cast onto an optically transparent substrate and measured by nondegenerate multiphoton microscopy.

**Fig. 6:**
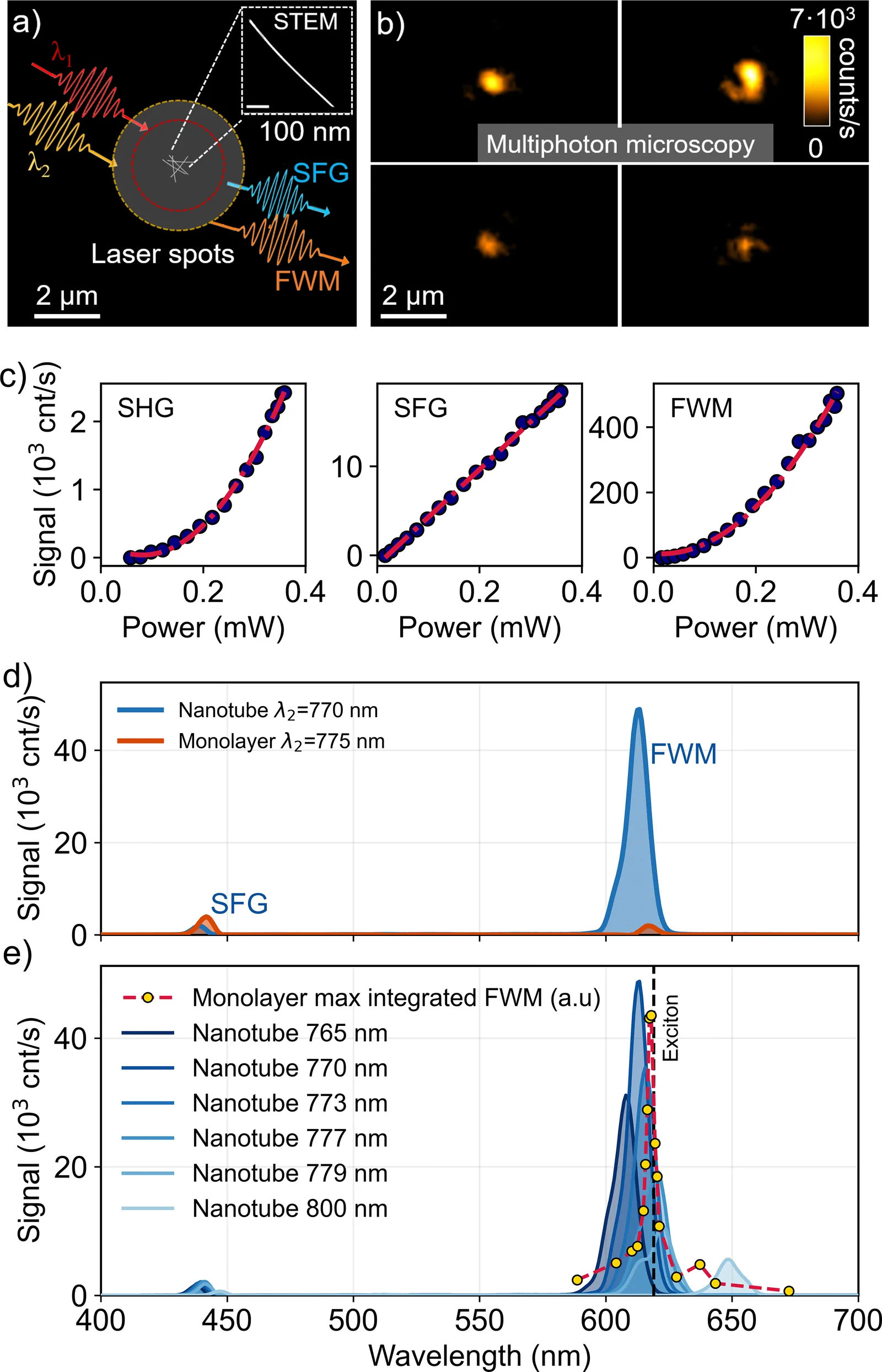
Nonlinear optical response from retained-core WS_2_ nanotubes grown from 100 nm W films. (a) Two-color excitation geometry and scale comparison with a representative retained-core nanotube. (b) Multiphoton microscopy images acquired at four nanotube-rich positions on the transparent substrate. (c) Power dependence of the SHG, SFG and FWM signals measured while varying *P*_*λ*_2__ and keeping *P*_*λ*_1__ constant; dashed-dotted curves show the expected nonlinear fits. (d) Raw detected nonlinear spectra from the nanotube and monolayer reference samples. (e) Wavelength-dependent nanotube FWM spectra obtained by tuning *λ*_2_; yellow markers indicate the scaled integrated monolayer FWM response used only to locate its spectral maximum. Scale bars: (a and b) 2 µm; STEM inset in (a): 100 nm.

Fig. 6a shows the two-color femtosecond laser excitation configuration, combining a fixed pulse at *λ*_1_ = 1030 nm with a synchronized tunable pulse in the range *λ*_2_ = 750–850 nm. For the representative measurements at *λ*_2_ = 770 nm, the beams were focused to 1/e^2^ diameters of *d*_1_ = 3.8 µm and *d*_2_ = 2.8 µm. This configuration allows the generated nonlinear wavelengths to be tuned relative to the excitonic response of WS_2_, including the monolayer exciton reference near 620 nm.^20,32^ Because the drop-cast nanotubes form assemblies rather than isolated objects, the detected signal originates from several nanotubes of varying length, diameter and orientation within the diffraction-limited focus, the measured response is therefore that of a nanotube assembly. The same optical platform was operated in both microscopy and spectroscopy modes, as described in Fig. S18.

Fig. 6b shows multiphoton microscopy images acquired at four nanotube-rich sample locations separated by several micrometres. The transferred nanotube assemblies exhibit localized nonlinear emission on the transparent substrate, whereas regions without nanotubes remain dark. Under comparable low-power imaging conditions, WS_2_ monolayers did not produce a measurable signal, motivating the use of a higher excitation power for the monolayer reference in the spectral comparison below. The two-color excitation scheme enables simultaneous detection of SHG, SFG, and FWM at the generated frequencies 2*ω*_2_, *ω*_1_ + *ω*_2_, and 2*ω*_2_ − *ω*_1_, respectively. For these nanoscale emitters, phase-matching constraints are relaxed relative to extended bulk crystals.

Fig. 6c shows the nonlinear optical signal as a function of the tunable-beam power at *λ*_2_ = 770 nm, while the *λ*_1_ = 1030 nm power is kept constant. The SHG, SFG, and FWM signals follow quadratic, linear, and quadratic dependencies on *P*_*λ*_2__, respectively, as expected for the assigned processes. This power-law behavior confirms the nonlinear origin of the detected spectral bands.

Fig. 6d compares the nonlinear spectra of the nanotube and monolayer samples, plotted as raw detected count rates. Even though the nanotube spectrum was acquired at a 20-fold lower tunable-beam power (*P*_*λ*_2__ = 20 µW *vs.* 400 µW), its FWM peak (∼50 × 10^3^ counts s^−1^) is more than an order of magnitude stronger than that of the monolayer reference (∼1.9 × 10^3^ counts s^−1^), a power-normalized estimate of this detected contrast is given in the SI (Section S11). We do not interpret this contrast as an intrinsic increase of *χ*^(3)^, because the two samples differ in illuminated material volume, wall number, orientation, optical anisotropy, local environment and overlap with the excitation focus, and because the nanotubes are measured as assemblies rather than isolated objects. Rather, it shows that these self-formed curved nanostructures are efficient nonlinear emitters, producing readily detectable SHG, SFG and FWM where planar WS_2_ is near the detection limit.

The nonlinear response is also spectrally resonant, as shown by the wavelength-dependent FWM data in Fig. 6e. Here the nanotube spectra are plotted as detected count rates, whereas the yellow markers show the integrated FWM signal from the monolayer reference, these monolayer values were not normalized to acquisition time and were scaled for visual comparison, so they are plotted in arbitrary units and used only to locate the spectral position of the monolayer FWM maximum (the amplitude comparison is based on the raw spectra in Fig. 6d). The strongest nanotube FWM response occurs near *λ*_FWM_ ≈ 613 nm, whereas the scaled monolayer reference peaks near *λ*_FWM_ ≈ 619 nm.^4,19,20,33^ This spectral shift indicates that the nonlinear response is resonantly mediated and that the relevant optical resonance is modified in the nanotube geometry: curvature, strain, reduced dimensionality, finite wall number and the retained-core geometry may alter the excitonic landscape of WS_2_, thereby modifying the resonant coupling between the FWM emission and excitonic states.

## 3. Conclusions

We have shown that the initial thickness of sputtered W films controls WS_2_ nanotube formation by defining the continuity, geometry, and conversion pathway of WO_*x*_-derived precursors during charcoal-assisted sulfurization. Across the thickness series, the same face-down charcoal-assisted growth geometry produces distinct morphology regimes. Ultrathin 1 nm W films favor laterally extended WS_2_-like domains, whereas 5 nm films break up into discontinuous WO_*x*_-rich domains that act as localized precursor reservoirs for sparse, hollow nanotubes with comparatively concentric shells. Increasing the thickness to 10 nm restores dense nanotube growth, but the nanotube shell structure becomes axially heterogeneous: locally converted regions show symmetric WS_2_ shells, while retained WO_*x*_-derived remnants are associated with diameter narrowing and local shell wrapping. At 100 nm, the precursor volume is sufficiently large that WO_*x*_-derived cores are retained over extended nanotube segments, leading to incomplete sulfurization, shell-front mismatch, and secondary shell wrapping.

These observations connect thin-film sulfurization with oxide-template nanotube conversion. Rather than acting only as a tungsten reservoir, the starting W film defines the oxide-derived precursor architecture that mediates nanotube nucleation, hollowing, and shell closure. Film thickness therefore provides a direct handle for selecting between laterally extended WS_2_-like domains, island-mediated nanotube nucleation, hollow concentric shell formation, and retained-core nanotube morphologies. This thickness-controlled conversion framework should be useful for designing substrate-supported WS_2_ nanotube networks and for understanding how oxide precursor continuity governs the topology of curved TMD nanostructures.

Finally, nondegenerate multiphoton microscopy shows that the nanotube assemblies obtained from the 100 nm growth regime are optically active nonlinear emitters, producing localized SHG, SFG and FWM that are readily detected under conditions where a WS_2_ monolayer reference is near the detection limit. We do not interpret this contrast as an intrinsic increase of *χ*^(3)^, because the nanotube and monolayer measurements differ in illuminated volume, wall number, orientation, local environment and collection efficiency, and because the nanotubes are measured as assemblies rather than isolated objects. The FWM resonance of the nanotubes is nonetheless spectrally shifted relative to the monolayer, indicating that the relevant excitonic resonance is modified in the nanotube geometry, curvature, finite wall number, strain, shell-front mismatch and the retained-core structure may all contribute, although the present measurements do not isolate any individual feature. Thickness-directed oxide-templated conversion therefore provides not only a route to controlling nanotube morphology but also self-formed curved-TMD structures with demonstrable nonlinear optical functionality, motivating their further study as building blocks for nonlinear nanophotonics.

## 4. Experimental

### 4.1. Materials

Tungsten foils, 0.05 mm thick and 99.95% purity, were purchased from Alfa Aesar and used as substrates for the W-foil control experiments. P-type Si(001) wafers were used as substrates for sputtered-W film samples.

### 4.2. Preparation of sputtered W films on Si

Thin W films were deposited on p-type Si(001) wafers using an alliance metal 1 DC magnetron sputtering system. Sputtering was performed from a polycrystalline W target in Ar at a pressure of 3.4 µbar and a power of 100 W. Before sputter deposition, the Si substrates were cleaned in fuming (100%) HNO_3_ for 7 min, followed by sonication in ethanol, acetone, isopropanol, and deionized water for 5 min each at high power, and then dried with N_2_. After loading the cleaned Si substrates into the chamber, the system was pumped to approximately 10^−7^ mbar before Ar was introduced. Before deposition, the target was isolated from the substrates with a shutter and pre-sputtered under the same conditions for 2 min to remove possible surface contaminants. Finally, the nominal W thickness was controlled by adjusting the sputtering time.

### 4.3. CVD sulfurization and sulfur-free precursor controls

WS_2_ nanotubes/nanostructures and sulfur-free WO_*x*_-derived precursor controls were prepared using an atmospheric-pressure CVD process in a three-zone horizontal gradient tube furnace (Carbolite Gero). Either 50 µm W foil or sputtered W films on Si were used as the W source, depending on the experiment. The substrate was mounted face-down above an alumina crucible containing charcoal powder and positioned in zone 2. For sulfurization experiments, sulfur powder was placed in a separate alumina boat in zone 3; for sulfur-free precursor controls, sulfur was omitted. After loading the substrate and precursors, the tube was purged with Ar at 1000 sccm for 30 min. Zone 2 was ramped at 10 °C min^−1^ to 640–700 °C and held for 1 s to 30 min, depending on the sample. During heating, the Ar flow was maintained at 1000 sccm until zone 2 reached 500 °C, after which it was reduced to 110–150 sccm for the remainder of the process. Zone 3 was either not independently heated or was ramped to 180–220 °C, depending on the experiment. After growth or annealing, samples were cooled naturally to room temperature under Ar. Detailed sample-specific conditions for the sputtered W/Si samples are summarized in Table S1, and the W-foil charcoal-control conditions are summarized in Table S2.

### 4.4. Sample preparation for TEM/STEM lamellae and transferred nanotubes

Cross-sectional lamellae were prepared by focused ion beam milling using a FEI Helios G4 CX dual-beam system. The lamella shown for the sulfur-free precursor analysis was prepared from a 5 nm W film on Si after charcoal-assisted CVD annealing without sulfur. For this sample, the substrate was loaded face-down above charcoal powder in zone 2, ramped to 700 °C, held for 1 s, and then cooled naturally to room temperature. Zone 3 was empty but set to 200 °C to maintain the thermal gradient used in the sulfurization experiments. After selecting the region of interest, a protective Pt layer of approximately 32 nm was first deposited by electron-beam-induced deposition at 86 pA and 10 kV. A thicker Pt layer of approximately 500 nm was then deposited by ion-beam-induced deposition at 7.7 pA and 30 kV. The Pt precursor was supplied using the gas-injection system, and lift-out was performed using an EasyLift tungsten micromanipulator. After transfer to a TEM grid, the lamella was thinned to electron transparency for TEM/STEM analysis. For plan-view nanotube STEM imaging and nanotube dimension measurements, nanotubes were transferred from the growth substrate onto TEM grids before analysis. Visibly broken, damaged, or strongly bent nanotubes were excluded from the length and diameter statistics.

### 4.5. Scanning electron microscopy

SEM images were acquired using a FEI NovaNano SEM equipped with a through-lens detector (TLD) operated in secondary electron mode, at accelerating voltages of 5 or 10 kV, depending on the sample.

### 4.6. Grazing-incidence X-ray diffraction

GIXRD measurements were performed using a Bruker Discover D8 diffractometer with Cu K*α* radiation, *λ* = 1.54 Å, at room temperature. The grazing-incidence angle was fixed at 0.2°. Diffractograms were acquired over a 2*θ* range of 10–80° with a step size of 0.02° and a step time of 0.2 s per step.

### 4.7. Raman spectroscopy

Raman spectra were acquired using a Renishaw inVia Reflex spectrometer at room temperature with a 514 nm excitation laser and a laser power of 5 mW. A Si wafer was used for calibration before each measurement.

### 4.8. Scanning transmission electron microscopy and STEM-EDX

Atomic-resolution HAADF-STEM and high-resolution STEM images of WS_2_ nanotubes were acquired using a probe-corrected JEOL ARM200F Mono microscope operated at 200 kV. The microscope was operated with the monochromator on, using a probe convergence semi-angle of 30.0 mrad. ADF-STEM imaging and STEM-EDX elemental mapping of the sulfur-free 5 nm precursor lamella were performed using a Titan Cube microscope operated at 300 kV.

STEM-EDX spectrum imaging was performed using a FEI Super-X G2 detector, with a dispersion of 10 eV per channel, an energy offset of −250 eV and a shaping time of 3 µs. The compositional maps were generated using the K family emission lines for C, O and S, and the L family lines for W and Pt. The STEM-EDX maps were used to identify the spatial distribution of W and O in the WO_*x*_-derived precursor domains.

### 4.9 Nonlinear optical microscopy and spectroscopy

Retained-core WS_2_ nanotubes grown from 100 nm W films were dispersed in isopropanol and drop-cast onto an optically transparent substrate. Nondegenerate multiphoton microscopy and spectroscopy were performed using a two-color femtosecond laser excitation scheme. A fixed pulse at *λ*_1_ = 1030 nm was combined with a synchronized tunable pulse in the range *λ*_2_ = 750–850 nm. The two beams were focused onto the sample using a high numerical-aperture objective, and the nonlinear emission was collected in the same optical path and directed either to a spectrometer or to a camera for imaging. Power-dependent measurements were acquired by varying the tunable-beam power while keeping the 1030 nm excitation power constant. The power-normalized FWM contrast was estimated from the raw detected peak count rates, accounting for the quadratic dependence of the FWM signal on the tunable-beam power. Full details of the optical setup are provided in the SI.

## Author contributions

C. S. synthesized the samples and carried out the CVD growth experiments, and performed the SEM, EDX, Raman and GIXRD characterization and the corresponding data analysis. S. C.-B. performed the STEM experiments. C. S. analyzed the electron microscopy data. J. H.-R. performed the nonlinear optical (multiphoton) measurements. J. H.-R., J. I. E. and D. J. D. analyzed the nonlinear optical data. S. C.-B. conceived and supervised the project. S. C.-B. wrote the manuscript with input from all authors. All authors contributed to the writing of the manuscript, discussed the results and approved the final version.

## Conflicts of interest

There are no conflicts to declare.

## Supplementary Material

TC-014-D6TC02147F-s001

## Data Availability

The data supporting the findings of this study are available within the article and its supplementary information (SI). Supplementary information: additional experimental and characterization data are provided. These include sample-specific CVD growth conditions, charcoal-assisted WO_*x*_ precursor-control experiments, spatial mapping of WO_*x*_ nanowire growth on W foil, additional SEM characterization of the sputtered W/Si thickness series, GIXRD and Raman analysis of tungsten oxide/suboxide precursor formation, STEM-EDX and HRTEM characterization of WO_*x*_-rich islands in 5 nm W films, extended HRSTEM analysis of WS_2_ nanotubes derived from 5, 10, and 100 nm W films, shell-front mismatch and spiral-like wrapping analysis, nanotube length/diameter statistics, W-foil charcoal-assisted precursor-control experiments, and nondegenerate multiphoton microscopy and spectroscopy setup. See DOI: https://doi.org/10.1039/d6tc02147f. The raw scanning electron microscopy (SEM), scanning transmission electron microscopy (STEM), STEM-energy-dispersive X-ray spectroscopy (STEM-EDX), Raman spectroscopy, grazing-incidence X-ray diffraction (GIXRD), and nonlinear optical microscopy and spectroscopy datasets generated during the current study are available from the corresponding author upon reasonable request.
