# Supplementary material for: Thickness-directed oxide-templated conversion of sputtered tungsten films into WS2 nanotubes
Source: J Mater Chem C Mater. 2026 Jul 30;14(35):16144–55. doi: 10.1039/d6tc02147f (PMC13439266; doi:10.1039/d6tc02147f)
Supplement: Supplementary file 1 [file TC-014-D6TC02147F-s001.pdf]

Supplementary Information (SI) for Journal of Materials Chemistry C.
This journal is © The Royal Society of Chemistry 2026

Supplementary Information for: Thickness-Directed Oxide-Templated Conversion of Sputtered Tungsten Films into WS_2 Nanotubes

C. Soukaras,[†] J. I. Echavarria,[‡,¶] D.J. Degeling,[†] J. Hernandez-Rueda,[‡,¶] and S. Conesa-Boj[*,†]

†*Kavli Institute of Nanoscience, Delft University of Technology, Delft, 2628 CJ, The Netherlands*

‡*Optics Department, Faculty of Physics, University Complutense of Madrid. Plaza de Ciencias 1, 28040 Madrid, Spain*

¶*Instituto de Química Física Blas Cabrera, IQF-CSIC, Serrano 119, 28006 Madrid, Spain*

E-mail: s.conesaboj@tudelft.nl

Contents

S1 CVD growth conditions and sample details 3

S2 Charcoal-assisted precursor control experiments 4

S3 Additional SEM images of W/Si thickness series 7

S4 Precursor formation in sputtered W/Si films 8

S5 Detailed indexing of the carbon-tape GIXRD reflections 9

S6 Cross-sectional STEM-EDX analysis of 5 nm precursor islands **14**

S7 Additional HRSTEM analysis of 5 nm W/Si nanotubes **16**

S8 Additional HRSTEM analysis of 10 nm W/Si nanotubes **18**

S9 HRSTEM analysis of 100 nm W/Si nanotubes **22**

S9.1 Retained-core morphology and nonuniform shell development 22

S9.2 Shell-front mismatch and spiral-like secondary wrapping 24

S10 Nanotube length and diameter statistics **26**

S11 Multiphoton microscopy of WS_2 nanotubes **28**

Power-normalized FWM enhancement estimate 29

References **31**

S1 CVD growth conditions and sample details

Table S1 summarizes the CVD growth conditions for all samples shown in the main manuscript and Supporting Information. The table includes the initial W source, growth time, substrate-zone temperature, sulfur-zone temperature, sulfur and charcoal loading, Ar flow and ramp rate. Unless otherwise stated, all samples were cooled naturally to room temperature under Ar after the growth step. The sulfur-free 5 nm W/Si experiment used for precursor analysis and the 1 s sulfurization experiment used to capture early nanotube nucleation are listed separately to distinguish precursor formation from sulfurization.

Table S1: CVD growth conditions for sputtered W/Si samples.

Sample	Figures	W [nm]	Time	T_{sub} [°C]	T_{S} [°C]	Notes
WNS-148	Fig. 1a, Fig. 5, Figs. S13–S16	100	30 min	700	n.h.	400.3 mg S; 52.1 mg C; 150 sccm Ar
WNS-151	Fig. 1b, Fig. 4, Figs. S4e–h, S11–S12	10	30 min	700	n.h.	406.7 mg S; 51.2 mg C; 110 sccm Ar
WNS-152	Fig. 1c, Fig. 3b–d, Figs. S4i–l, S9–S10	5	30 min	700	n.h.	402.0 mg S; 53.7 mg C; 110 sccm Ar
WNS-153	Fig. 1d, Figs. S4m–p	1	30 min	700	n.h.	400.3 mg S; 46.8 mg C; 110 sccm Ar
WNS-162	Fig. 2, Figs. S5b, S6–S8	5	1 s	700	200	sulfur-free; 51.8 mg C; 110 sccm Ar
WNS-158	Fig. 3a	5	1 s	700	200	502.8 mg S; 51.0 mg C; 110 sccm Ar
WNS-147	Figs. S4a–d	100	30 min	700	n.h.	402.6 mg S; 45.5 mg C; 110 sccm Ar

n.h. = sulfur zone not independently heated. T_{sub} is the substrate-zone set temperature and T_{S} is the sulfur-zone set temperature. Unless otherwise stated, the ramp rate was 10 °C min^{-1}, and samples were cooled naturally under Ar after growth. S and C indicate the loaded sulfur and charcoal masses, respectively.

S2 Charcoal-assisted precursor control experiments

To evaluate the role of charcoal in WO_x-derived precursor formation, we performed control experiments on W foil under sulfur-free CVD conditions. The CVD conditions for these W-foil control samples are summarized in Table S2. In the first approach, the active surface of the W foil was briefly contacted with carbon tape and the tape was then removed, leaving a thin carbonaceous surface residue before CVD processing (Fig. S1a). In the second approach, charcoal powder was placed beneath the face-down W foil during CVD processing, without direct contact between the charcoal and the foil surface (Fig. S1b). These two configurations allow us to distinguish the effect of a residual surface carbon layer from that of the local charcoal-assisted environment beneath the substrate.

Table S2: CVD conditions for W-foil charcoal-control experiments.

Sample	Figures	W source	Time	T_{sub} [°C]	Notes
WNS-109	Fig. S1a, Fig. S6 solid curve	W foil, 50 μm	30 min	640	no sulfur; no charcoal; 110 sccm Ar
WNS-110	Fig. S1b	W foil, 50 μm	30 min	640	no sulfur; 48.5 mg charcoal; 110 sccm Ar
WNS-111	Figs. S2–S3	W foil, 50 μm	30 min	640	no sulfur; 48.4 mg charcoal; 110 sccm Ar
WNS-137	Fig. S6 dashed curve	W foil, 50 μm	30 min	640	no sulfur; 51.6 mg charcoal; 110 sccm Ar

Unless otherwise stated, the sulfur zone was not independently heated, the ramp rate was 10 °C min^{-1}, and samples were cooled naturally under Ar.

Only sparse, short nanowires are observed after brief carbon-tape contact (Fig. S1a). By contrast, the charcoal-assisted configuration produces a higher density of longer WO_x nanowires (Fig. S1b). This comparison indicates that charcoal powder placed beneath the face-down W foil promotes WO_x-derived nanowire precursor formation more effectively than a residual carbonaceous surface layer.

We next examined whether the charcoal-assisted effect is spatially localized. For this experiment, the W foil was placed face-down above the charcoal mount, with part of the foil extending beyond the crucible rims. Because the foil is longer than the alumina crucible,

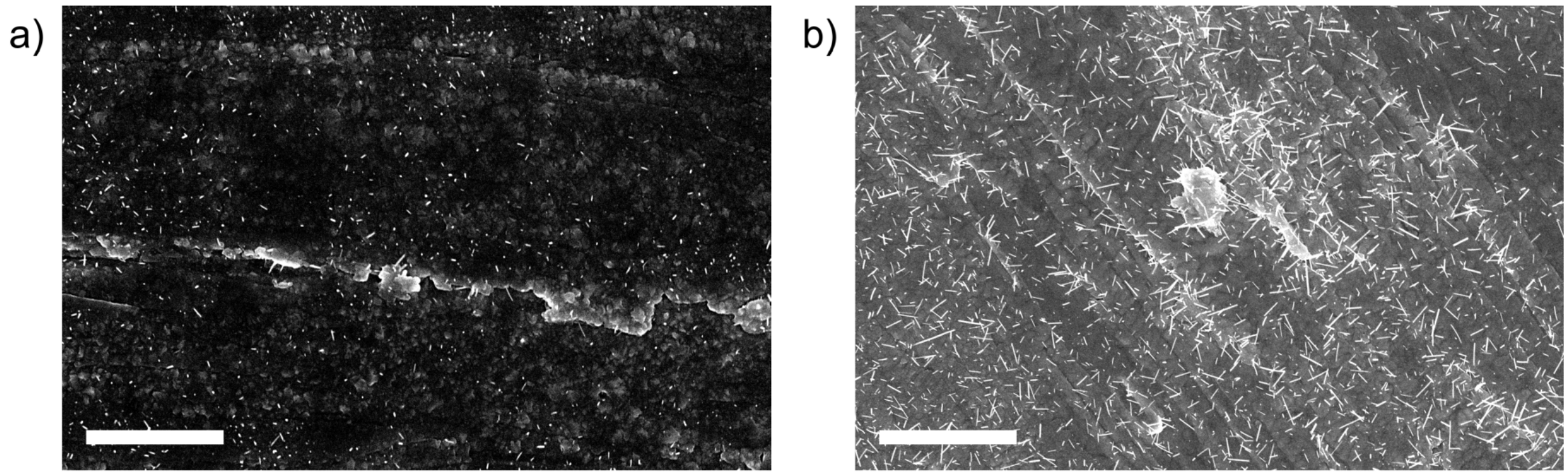

Figure S1: **Effect of carbon source on WO_x nanowire growth on W foil. (a)** SEM image of the W foil surface after brief carbon-tape contact followed by sulfur-free CVD processing, showing sparse and short WO_x nanowires. **(b)** SEM image of the W foil surface processed under the same sulfur-free CVD conditions but with charcoal powder placed beneath the face-down substrate, showing a higher density of longer WO_x nanowires. Scale bars: 2 μm.

only the central region is positioned directly above the charcoal powder. SEM images were acquired at five locations from the center of the foil to the edge (Fig. S2). Dense, highly uniform nanowires with high aspect ratios are observed in regions near the center of the foil, directly above the charcoal mount (Fig. S2a,b). A pronounced reduction in nanowire density is observed at the transition region where the foil begins to extend beyond the crucible rims (Fig. S2c). At locations close to the foil edge and corner, farther from the charcoal mount, no nanowires are observed (Fig. S2d,e). The corresponding macroscopic image and schematic of the crucible–foil geometry are shown in Fig. S3.

This spatial dependence is also visible to the naked eye as a color contrast on the W foil, with a clear boundary separating the region that was positioned above the charcoal mount from the outer regions beyond its reach (Fig. S3).

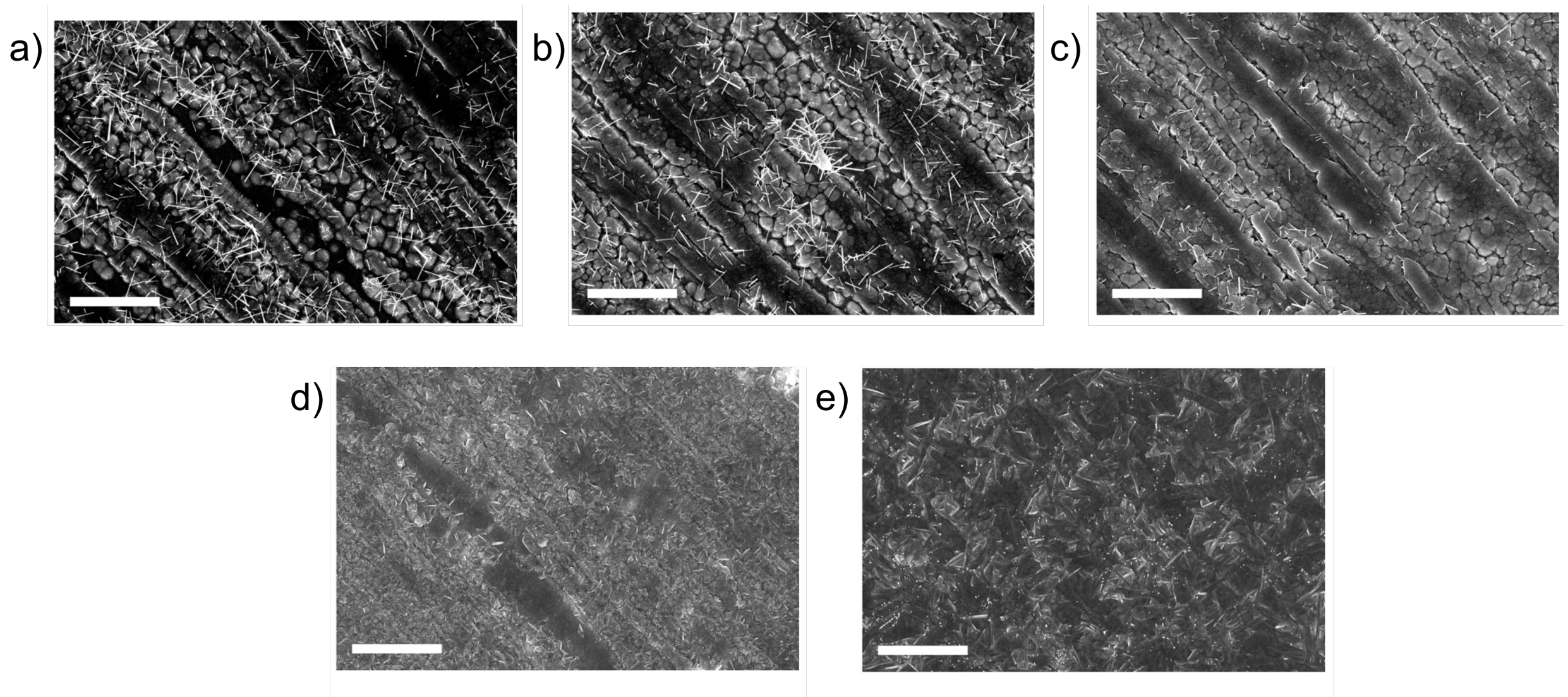

Figure S2: **Spatial mapping of WO_x nanowire growth on W foil under charcoal-assisted CVD.** SEM images were acquired at different positions along a W foil processed under sulfur-free CVD conditions with charcoal powder placed beneath the face-down substrate. **(a)** Central region of the foil, directly above the charcoal mount, showing a dense and uniform WO_x nanowire network. **(b)** Intermediate region, still showing high nanowire density and spatial uniformity. **(c)** Boundary region where the foil begins to extend beyond the crucible rims, showing a pronounced decrease in nanowire density. **(d,e)** Edge and corner regions farther from the charcoal mount, where no nanowires are observed. Scale bars: 2 μm.

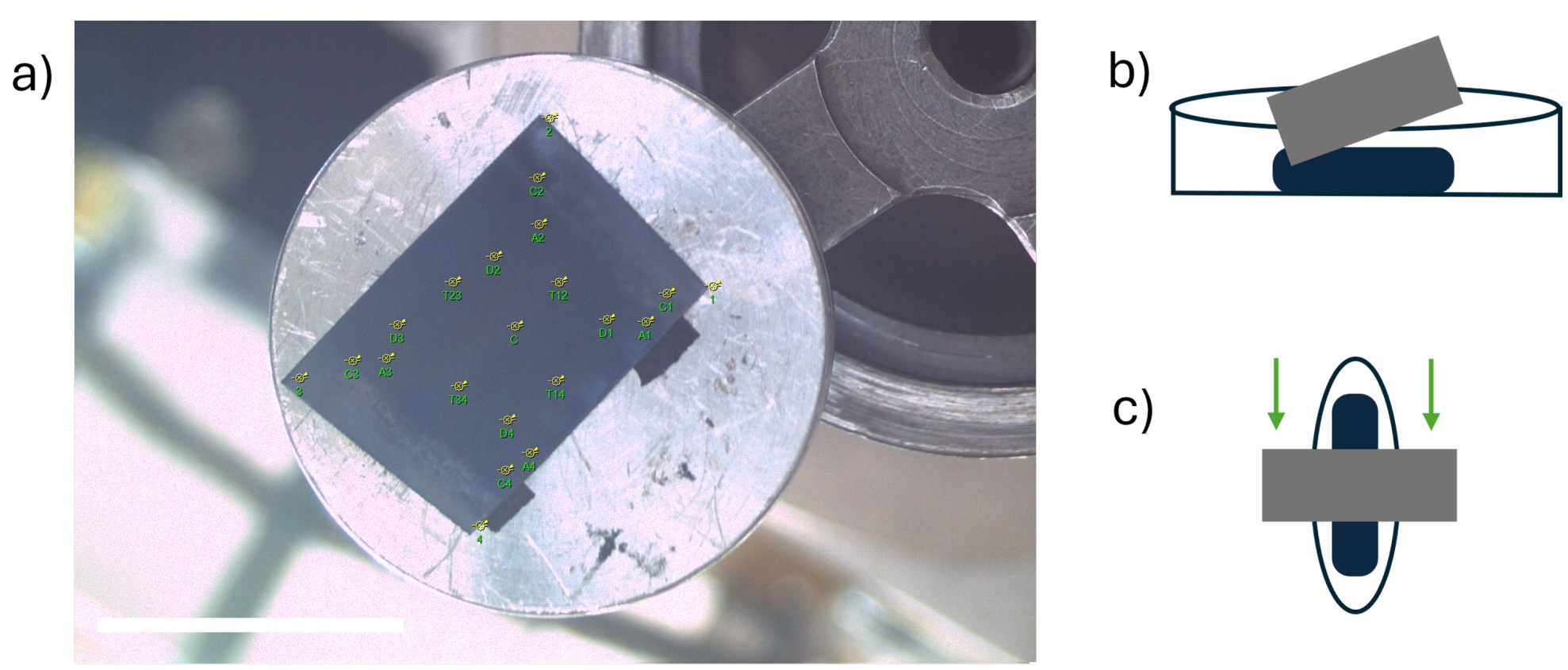

Figure S3: **Macroscopic image and schematic of the local charcoal-assisted effect on WO_x nanowire growth.** **(a)** Navigation-camera image of the W foil after sulfur-free charcoal-assisted CVD processing, showing a color contrast between the central region positioned above the charcoal mount and the outer foil regions extending beyond the crucible rims. **(b)** Side-view schematic of the alumina crucible geometry, showing charcoal powder placed beneath the face-down W foil. **(c)** Top-view schematic of the crucible–foil geometry. The highlighted outer regions indicate parts of the W foil positioned farther from the charcoal mount, where reduced or absent nanowire growth is observed. Scale bar in **(a)**: 10 mm.

S3 Additional SEM images of W/Si thickness series

Additional SEM images were acquired from spatially distinct regions of each sulfurized W/Si substrate to evaluate the consistency of the thickness-dependent morphologies described in the main text. The images shown in Fig. S4 support the progression from dense nanotube-rich growth for thicker W films, to sparse island-associated nanotubes in the 5 nm regime, and predominantly laterally extended WS_2 domains for the 1 nm film.

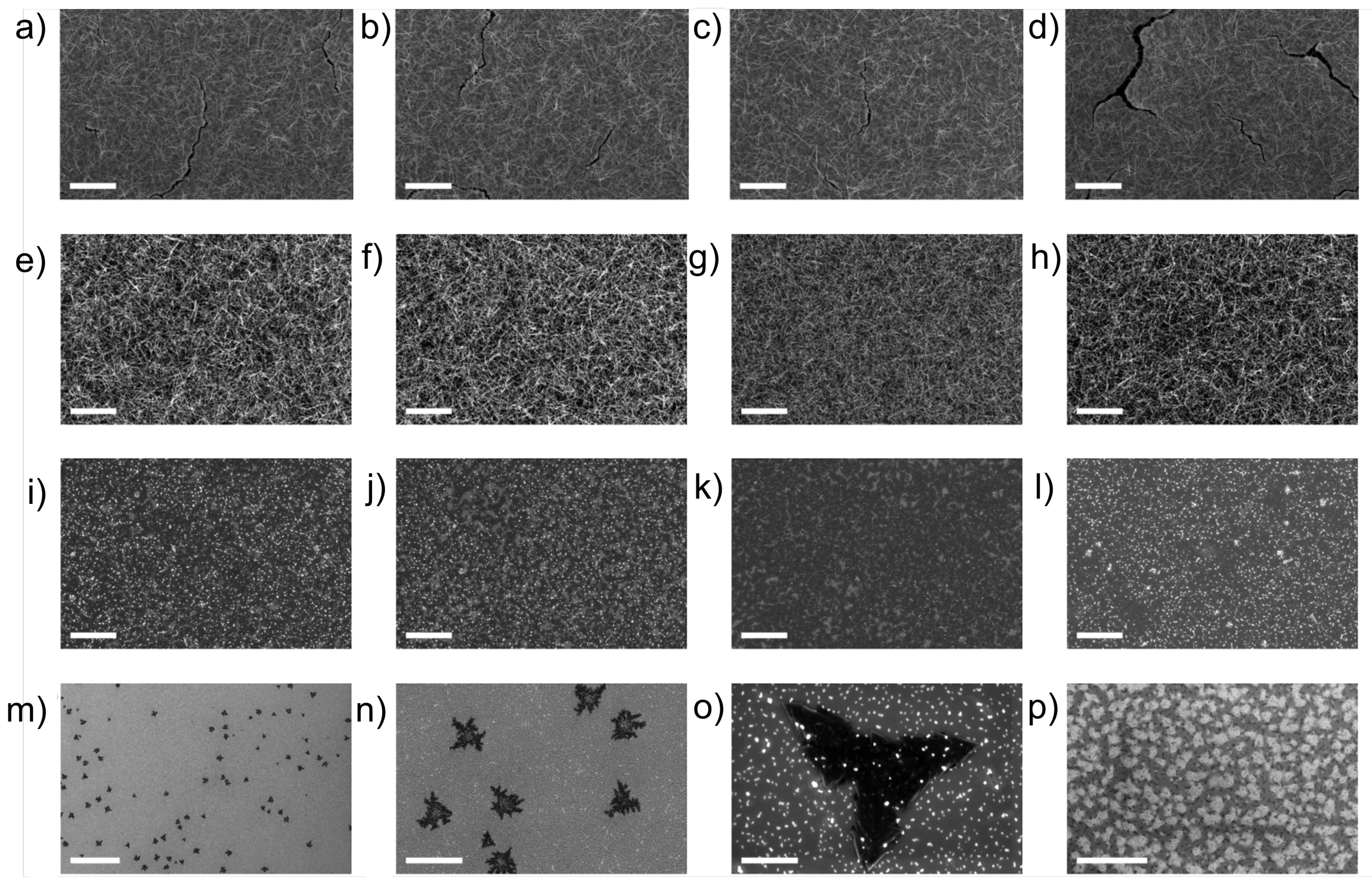

Figure S4: **Additional SEM images of thickness-dependent WS_2 morphologies grown from sputtered W/Si substrates.** SEM images acquired from spatially distinct regions of substrates with initial W thicknesses of **(a)-(d)** 100 nm, **(e)-(h)** 10 nm, **(i)-(l)** 5 nm, and **(m)-(p)** 1 nm. The 100 and 10 nm films show dense nanotube-rich morphologies across the sampled regions. The 5 nm film shows a sparser nanotube population associated with island-like domains and laterally grown WS_2 regions. The 1 nm film predominantly forms laterally extended WS_2 domains with residual island-like features, indicating suppression of nanotube-rich growth at the lowest W thickness. Scale bars: **(a–l)** 5 μm; **(m)** 100 μm; **(n)** 20 μm; **(o)** 2 μm; **(p)** 10 μm.

S4 Precursor formation in sputtered W/Si films

To examine the precursor stage before sulfurization, a 5 nm W/Si sample was processed under the same charcoal-assisted CVD geometry used for nanotube growth, but without sulfur. Before annealing, the sputtered W film appears smooth and laterally continuous by SEM (Fig. S5a). After sulfur-free charcoal-assisted annealing, the surface reorganizes into a heterogeneous morphology containing island-like domains and nanowire-like structures (Fig. S5b). These observations show that discontinuous oxide-derived precursor structures can form from the 5 nm W film before WS_2 conversion.

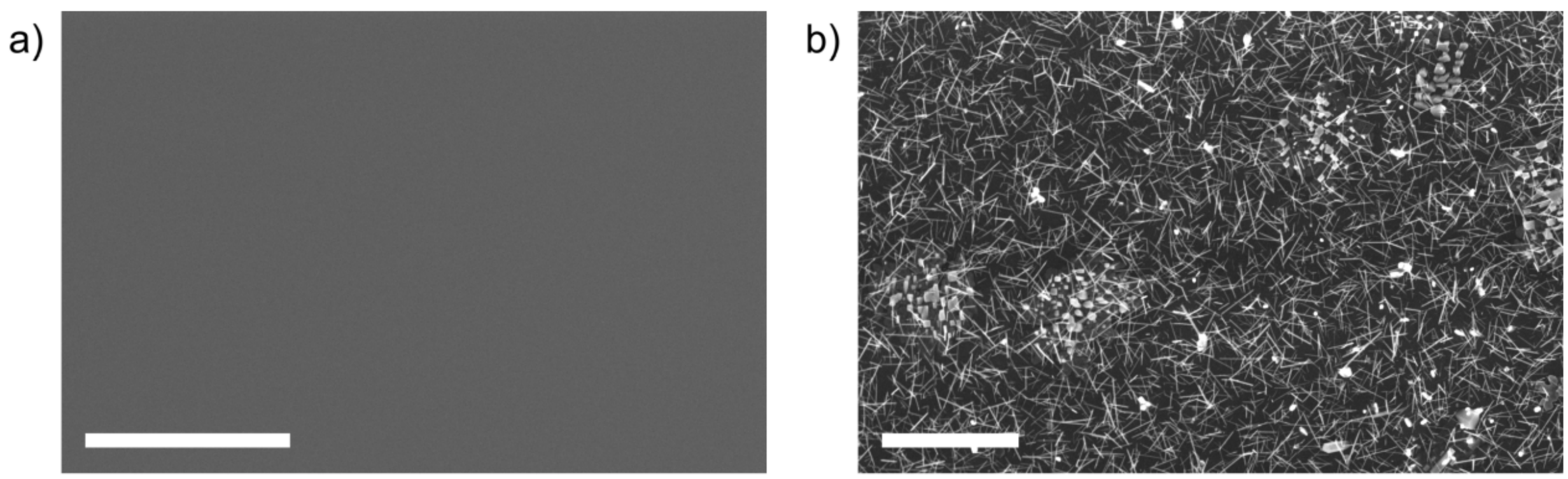

Figure S5: **Sulfur-free precursor formation in charcoal-assisted CVD of 5 nm W/Si.** **(a)** SEM image of the as-deposited 5 nm W/Si film before CVD annealing, showing a comparatively smooth surface. **(b)** SEM image after charcoal-assisted CVD annealing without sulfur, showing island-like domains and nanowire-like structures assigned to WO_x-derived precursor formation. Scale bars: **(a)** 3 μm; **(b)** 2 μm.

Grazing-incidence X-ray diffraction (GIXRD) and Raman spectroscopy were used to compare the structural evolution of W foil processed under carbon-tape and charcoal-assisted conditions, and to characterize the sulfur-free 5 nm W/Si precursor sample (Fig. S6). The extracted peak positions are summarized in Tables S3 and S5. Because tungsten oxide and suboxide reference patterns contain overlapping diffraction and vibrational features, the assignments are treated as phase-consistent rather than unique.

Table S3: GIXRD peak positions measured for W foil and 5 nm W/Si samples before and after sulfur-free CVD processing. Reference assignments are provided for comparison only; tungsten oxide and suboxide phases have overlapping diffraction features, so the assignments are treated as phase-consistent rather than unique.

**Sample**	**Measured 2θ [deg]**	**Tentative assignment / comment**
Pristine W foil	40.49, 58.28, 73.24	Cubic W reflections assigned to W (110), W (200), and W (211), respectively; reference values: 40.2, 58.2, and 73.1°.[1]
W foil annealed with carbon tape	23.32, 23.67, 28.69, 33.47, 48.25, 50.58, 59.70, 73.88	Prominent reflections consistent with tungsten oxide/suboxide-related phases, including a double reflection at 23.32/23.67°, with intensities exceeding those of the retained W reflections. Each reflection is compatible with reported reflections of several WO_3 polymorphs and of substoichiometric $WO_{2.72}$; the detailed reflection-by-reflection correlation with JCPDS reference cards is provided in Table S4. The peak at 73.88° may overlap with W/WO_x-related contributions, in particular with the W (211) reflection (JCPDS 04-0806).
W foil annealed with charcoal powder	40.88, 74.12	Retained W reflections assigned to W (110) and W (211), respectively. Oxide-related reflections are weak or below the detection limit under these conditions.
5 nm W/Si, sulfur-free charcoal anneal	14.14, 56.05	Weak reflections consistent with tungsten suboxide-related phases; reference values near 14.5 and 56.3° are reported for reduced tungsten oxide/suboxide phases.[2]

S5 Detailed indexing of the carbon-tape GIXRD reflections

Table S4 provides the reflection-by-reflection correlation of the GIXRD peaks measured for the W foil annealed with carbon tape (Table S3 and Fig. S6a) with JCPDS reference cards of tungsten oxide, suboxide and metallic W phases. Several WO_3 polymorphs and substoichiometric $WO_{2.72}$ exhibit closely overlapping reflections at each measured angle; for example, the prominent double reflection at 23.32/23.67° is compatible with the monoclinic, orthorhombic and tetragonal WO_3 polymorphs as well as with $WO_{2.72}$. This multiplicity is consistent with the thermal history of the samples, since different WO_3 polymorphs are

stable at the annealing temperature and upon cooling to room temperature. Accordingly, the assignments in Table S4 are treated as phase-consistent rather than unique.

Table S4: Reflection-by-reflection correlation of the GIXRD peaks measured for the W foil annealed with carbon tape with JCPDS reference cards of tungsten oxide, suboxide and metallic W phases. Several phases exhibit overlapping reflections at each measured angle, so the assignments are phase-consistent rather than unique.

**Measured 2θ [deg]**	**Tentative assignment**	**Reference**
23.32	Monoclinic WO_3 (002)	JCPDS 83-0950
	Hexagonal WO_3 (110)	JCPDS 75-2187
	Orthorhombic WO_3 (001)	JCPDS 20-1324
	Tetragonal WO_3 (001)	JCPDS 89-1287
	$WO_{2.72}$ (010)	JCPDS 05-0392
23.67	Monoclinic WO_3 (020)	JCPDS 83-0950
	Orthorhombic WO_3 (020)	JCPDS 20-1324
	Tetragonal WO_3 (200)	JCPDS 89-1287
28.69	Hexagonal WO_3 (200)	JCPDS 75-2187
	Monoclinic WO_3 (112)	JCPDS 83-0950
	Orthorhombic WO_3 (111)	JCPDS 20-1324
33.47	Hexagonal WO_3 (111)	JCPDS 75-2187
	Monoclinic WO_3 (022)	JCPDS 83-0950
	Orthorhombic WO_3 (021)	JCPDS 20-1324
	Tetragonal WO_3 (201)	JCPDS 89-1287
48.25	Monoclinic WO_3 (232)	JCPDS 83-0950
	Hexagonal WO_3 (220)	JCPDS 75-2187
	$WO_{2.72}$ (020)	JCPDS 05-0392
50.58	Tetragonal WO_3 (400)	JCPDS 89-1287
	Monoclinic WO_3 (400)	JCPDS 83-0950
	Orthorhombic WO_3 (400)	JCPDS 20-1324
59.70	Orthorhombic WO_3 (414)	JCPDS 20-1324
	Tetragonal WO_3 (421)	JCPDS 89-1287
73.88	Cubic W (211)	JCPDS 04-0806
	Tetragonal WO_3 (332)	JCPDS 89-1287

The GIXRD pattern of pristine W foil shows the expected cubic W reflections. After sulfur-free annealing with carbon-tape contact, prominent reflections appear that are consistent with tungsten oxide or suboxide phases, including a double reflection at 23.32/23.67°, with intensities exceeding those of the retained W reflections. In the charcoal-assisted W-foil

Table S5: Raman peak positions and tentative vibrational assignments for W foil and 5 nm W/Si samples after sulfur-free CVD processing. The δ and ν symbols denote bending/deformation and stretching modes, respectively. Assignments are based on reported tungsten oxide/suboxide Raman modes and should be interpreted together with the GIXRD and microscopy data.

**Sample**	**Raman shift [cm^{-}1]**	**Tentative vibrational assignment**
W foil annealed with charcoal powder	284.5	δ (O–W–O)
	807.3	ν (O–W–O)
	1364.0	carbon D band
W foil annealed with carbon tape	185.8	lattice vibration
	261.6	δ (O–W–O)
	299.0	δ (O–W–O)
	673.7	ν (O–W–O)
	811.4	ν (O–W–O)
5 nm sputtered W/Si	262.2	δ (O–W–O)
	326.1	δ (O–W–O)
	520.8	Si substrate
	708.3	ν (O-W-O)
	802.1	ν (O-W-O)
	898.9	ν (W=O)
	~970.0	Si substrate

sample, strong W reflections remain visible, while oxide-related reflections are weak or below the detection limit. The retained W reflections of the charcoal-annealed foil appear shifted to higher angles relative to the pristine W reference (40.88 vs 40.49° and 74.12 vs 73.24°). The shift increases with diffraction angle approximately as $\tan\theta$, as expected for a uniform reduction of the interplanar spacings, and corresponds to a relative contraction of approximately 1%. This behavior is consistent with compressive strain of the W lattice and/or incorporation of light elements during charcoal-assisted annealing, rather than with an instrumental sample-displacement effect, which would produce a shift decreasing with angle. For the sulfur-free 5 nm W/Si sample, weak reflections at approximately $2\theta = 14.14°$ and 56.05° are consistent with tungsten suboxide-related phases, although the overlap among tungsten oxide/suboxide reference patterns prevents unambiguous phase assignment from GIXRD alone.

Raman measurements further support tungsten oxide/suboxide formation. The carbon

tape and charcoal-assisted W-foil samples show O-W-O bending/deformation and stretching modes, with peak positions listed in Table S5. The charcoal-assisted W-foil spectrum also shows a feature near 1364 cm^{-1}, consistent with a carbon D-band contribution from residual carbonaceous species. For the sulfur-free 5 nm W/Si sample, Raman bands at 262.2 and 326.1 cm^{-1}, together with bands at 708.3 and 802.1 cm^{-1}, are consistent with O–W–O bending/deformation and stretching modes. A weaker feature near 898.9 cm^{-1} is consistent with W=O stretching contribution, while the peaks at 520.8 and 970 cm^{-1} arise from the silicon substrate. Taken together, SEM, GIXRD, and Raman data support the formation of WO_x-derived precursor structures during sulfur-free charcoal-assisted annealing of 5 nm W/Si.

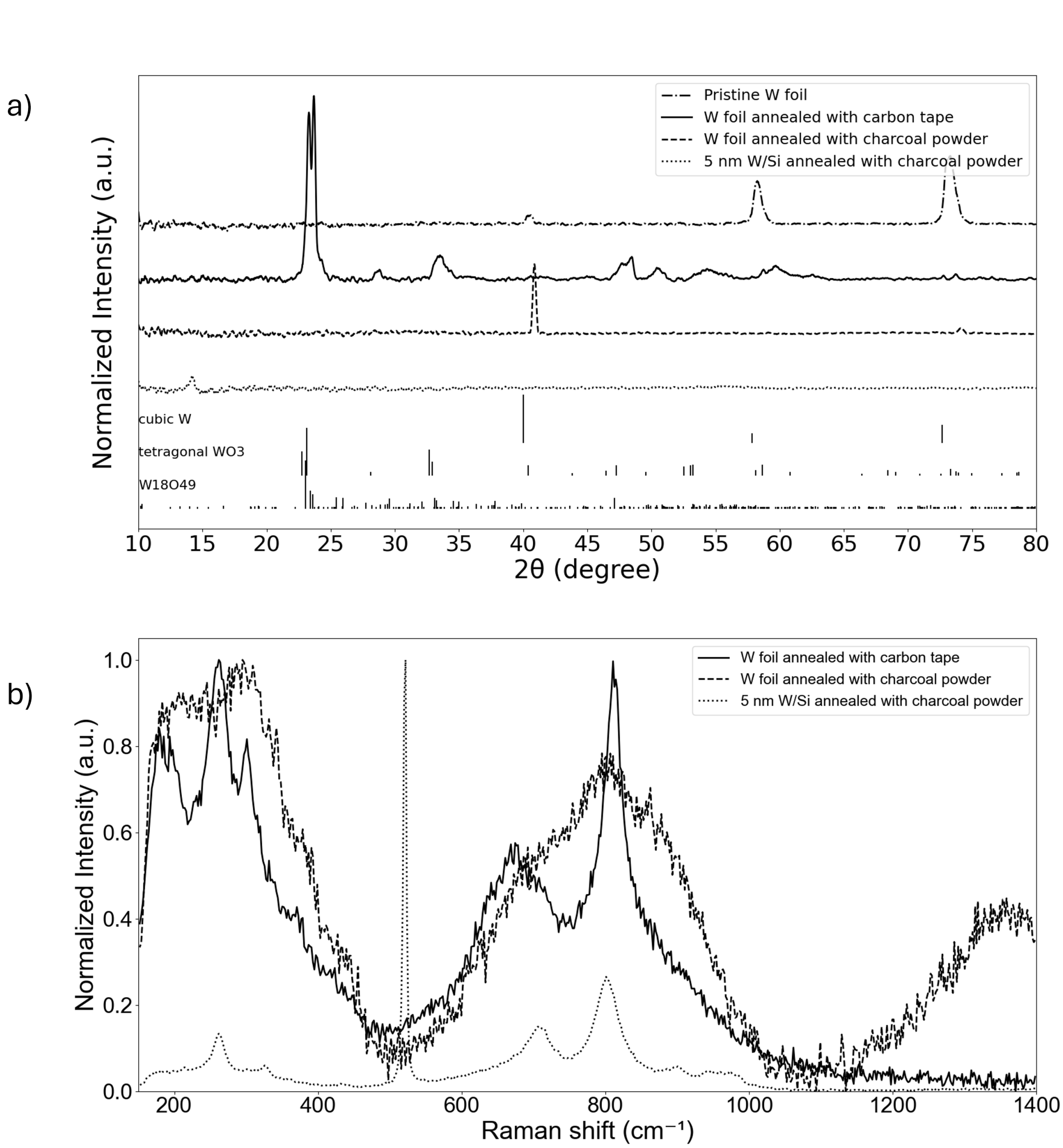

Figure S6: **GIXRD and Raman characterization of tungsten oxide/suboxide precursor formation.** **(a)** Normalized GIXRD patterns of pristine W foil, W foil after sulfur-free CVD annealing with carbon-tape contact, W foil after sulfur-free charcoal-assisted CVD annealing, and 5 nm W/Si after sulfur-free charcoal-assisted CVD annealing. Reference peak positions for cubic W, WO_3, and $W_{18}O_{49}$ are included for comparison. **(b)** Raman spectra of the corresponding processed samples. The spectra show O–W–O bending/deformation and stretching modes characteristic of tungsten oxide/suboxide species, together with Si substrate features for the 5 nm W/Si sample. Peak positions and tentative assignments are summarized in Tables S3 and S5.

S6 Cross-sectional STEM-EDX analysis of 5 nm precursor islands

To investigate the structure and elemental composition of the discontinuous precursor domains formed during sulfur-free charcoal-assisted annealing, a cross-sectional lamella was prepared from the 5 nm W/Si precursor-control sample. The lamella was taken through a region containing several island-like domains, including faceted and irregularly shaped features. A low-magnification TEM overview and selected high-resolution regions are shown in Fig. S7.

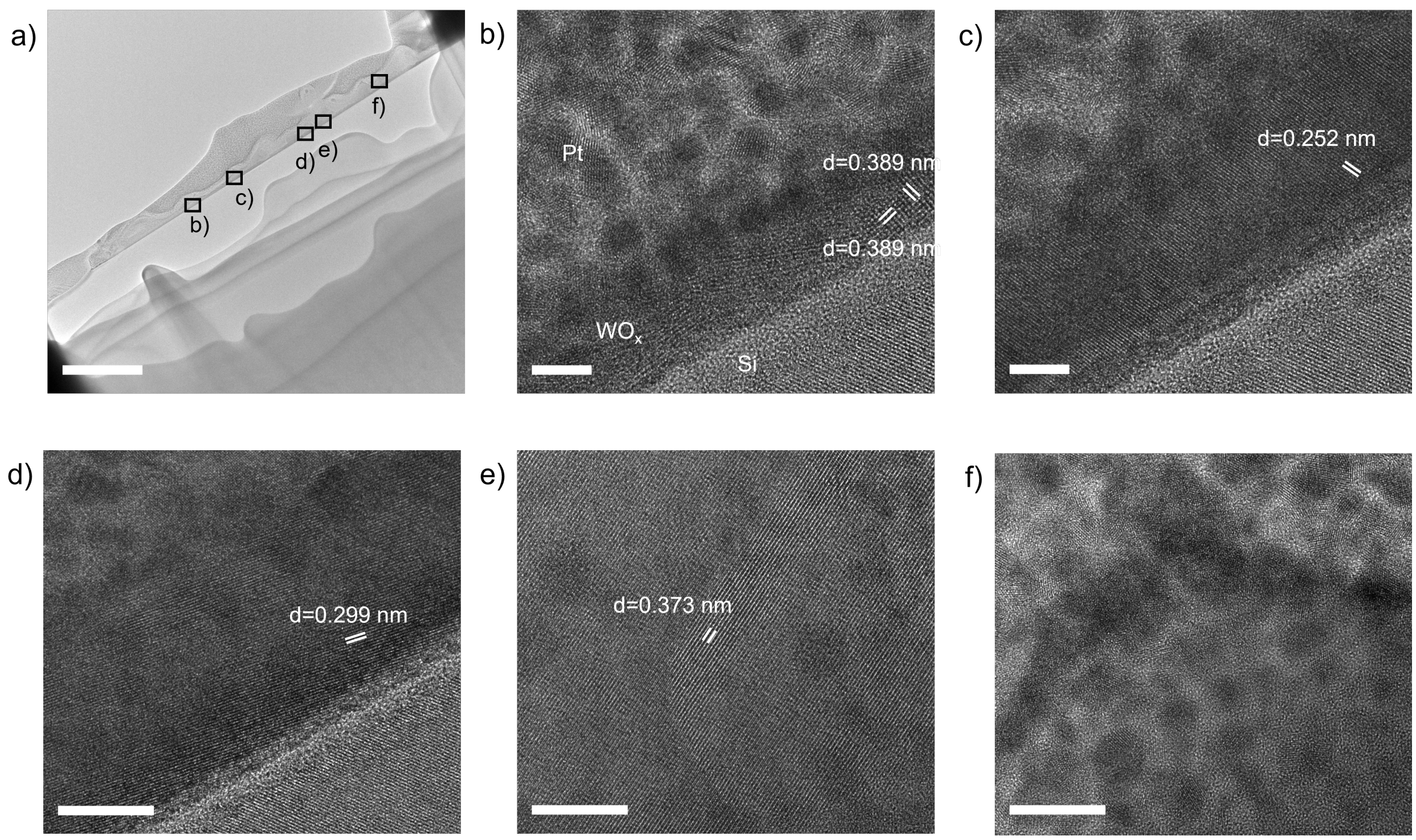

Figure S7: **Cross-sectional HRTEM characterization of WO_x-derived precursor domains in the sulfur-free 5 nm W/Si sample.** **(a)** Low-magnification TEM image of the FIB-prepared lamella, with selected regions marked for higher-resolution analysis. **(b–f)** HRTEM images showing variable precursor-layer thickness, local crystalline domains and amorphous material. Representative lattice spacings of approximately 0.252, 0.299 and 0.373 nm are measured in panels **(c–e)**, respectively, consistent with tungsten oxide/suboxide-related domains but not sufficient for unique phase assignment. Scale bars: **(a)** 500 nm; **(b)-(c)** 5 nm; **(d)-(f)** 10 nm.

The cross-sectional images show that the initially nominal 5 nm W film does not remain uniform after sulfur-free annealing. Instead, the W-derived layer reorganizes into regions with variable thickness, local crystalline domains and amorphous material. In one region, a comparatively uniform layer remains close to the nominal film thickness, while a darker island-like feature with a height of approximately 15 nm forms above it (Fig. S7c). The measured lattice spacing in this feature is approximately 0.252 nm, consistent with a tungsten oxide/suboxide-related crystalline domain. In another region, the crystalline contrast is oriented nearly parallel to the substrate surface and gives an interplanar spacing of approximately 0.299 nm (Fig. S7d). A faceted island-like domain near the W/Si interface shows a larger spacing of approximately 0.373 nm (Fig. S7e). These spacings are consistent with WO_x-related phases, but the overlap among tungsten oxide and suboxide lattice spacings prevents assignment of a unique crystallographic phase from HRTEM alone.

The local increase in thickness relative to the nominal 5 nm sputtered W film indicates redistribution and agglomeration of W-derived material during the sulfur-free charcoal-assisted annealing step. This behaviour is consistent with the SEM evidence in Fig. S5, where the initially smooth film reorganizes into island-like domains and nanowire-like structures before sulfurization.

Elemental mapping further supports the WO_x-derived nature of these precursor domains. STEM-EDX maps acquired from an island-containing region show W and O enrichment in the film and island-like domains (Fig. S8). A nonuniform W/O-containing layer is observed above the Si substrate, with local thickness variations reaching approximately 20 nm. The sulfur signal is negligible, consistent with the sulfur-free processing condition and minimal cross-contamination from previous CVD experiments. Carbon is detected locally, most likely from residual carbonaceous species and/or sample-preparation contributions, while weak Cu signal originates from the TEM grid. Together, the HRTEM and STEM-EDX data support the assignment of the sulfur-free 5 nm precursor morphology to discontinuous WO_x-derived domains.

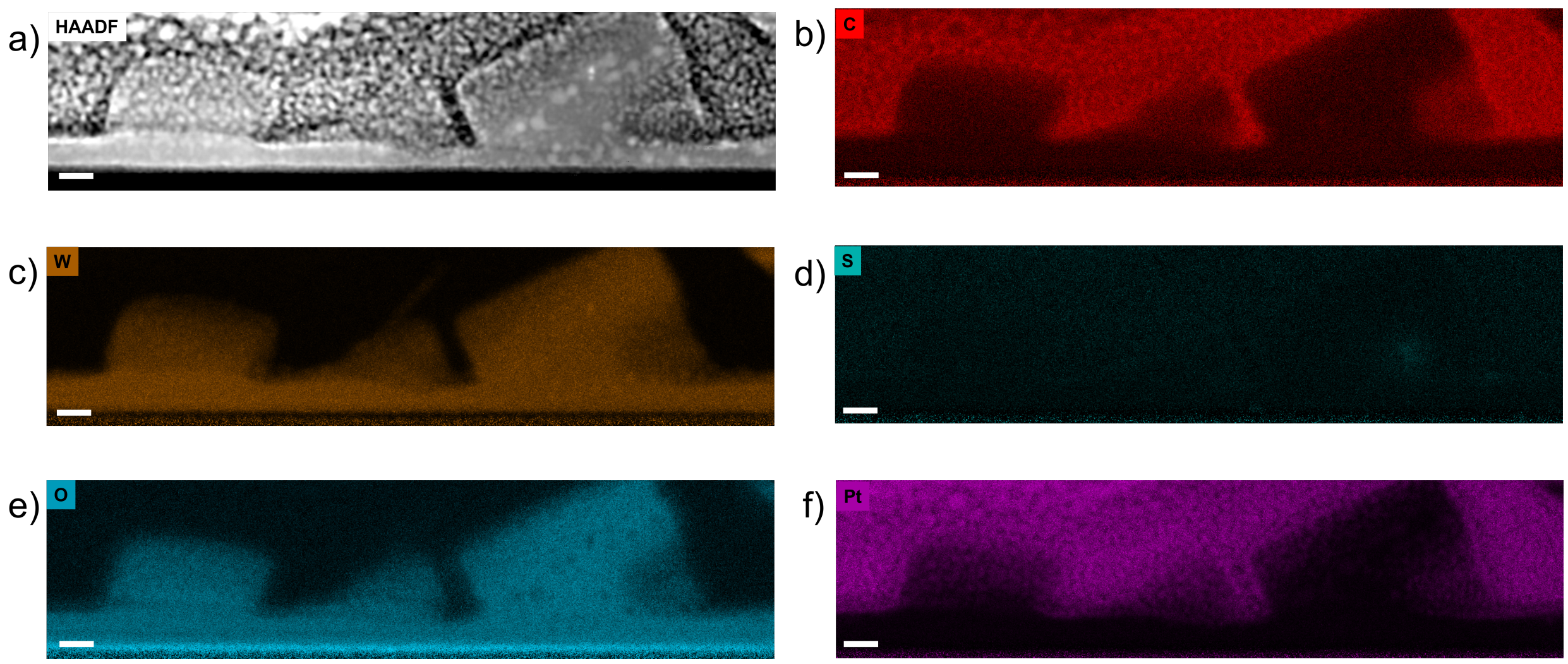

Figure S8: **STEM-EDX analysis of W/O-rich precursor domains in the sulfur-free 5 nm W/Si sample.** HAADF-STEM image of an island-containing lamella region with corresponding STEM-EDX maps for S, C, W, Pt and O. The island-like domains and nonuniform film region are enriched in W and O, supporting their assignment as WO_x-derived precursor structures. The weak sulfur signal is consistent with the sulfur-free annealing condition, while Pt and Cu-related signals arise from lamella preparation and the TEM grid. Scale bars: 20 nm.

S7 Additional HRSTEM analysis of 5 nm W/Si nanotubes

Additional HRSTEM images were acquired to evaluate the shell structure of WS_2 nanotubes grown from 5 nm W/Si. The images show hollow nanotube segments with concentric multilayer WS_2 walls over extended regions of the nanotube length. Local variations in wall thickness and shell continuity are occasionally observed, but the concentric shell morphology is recovered away from these regions.

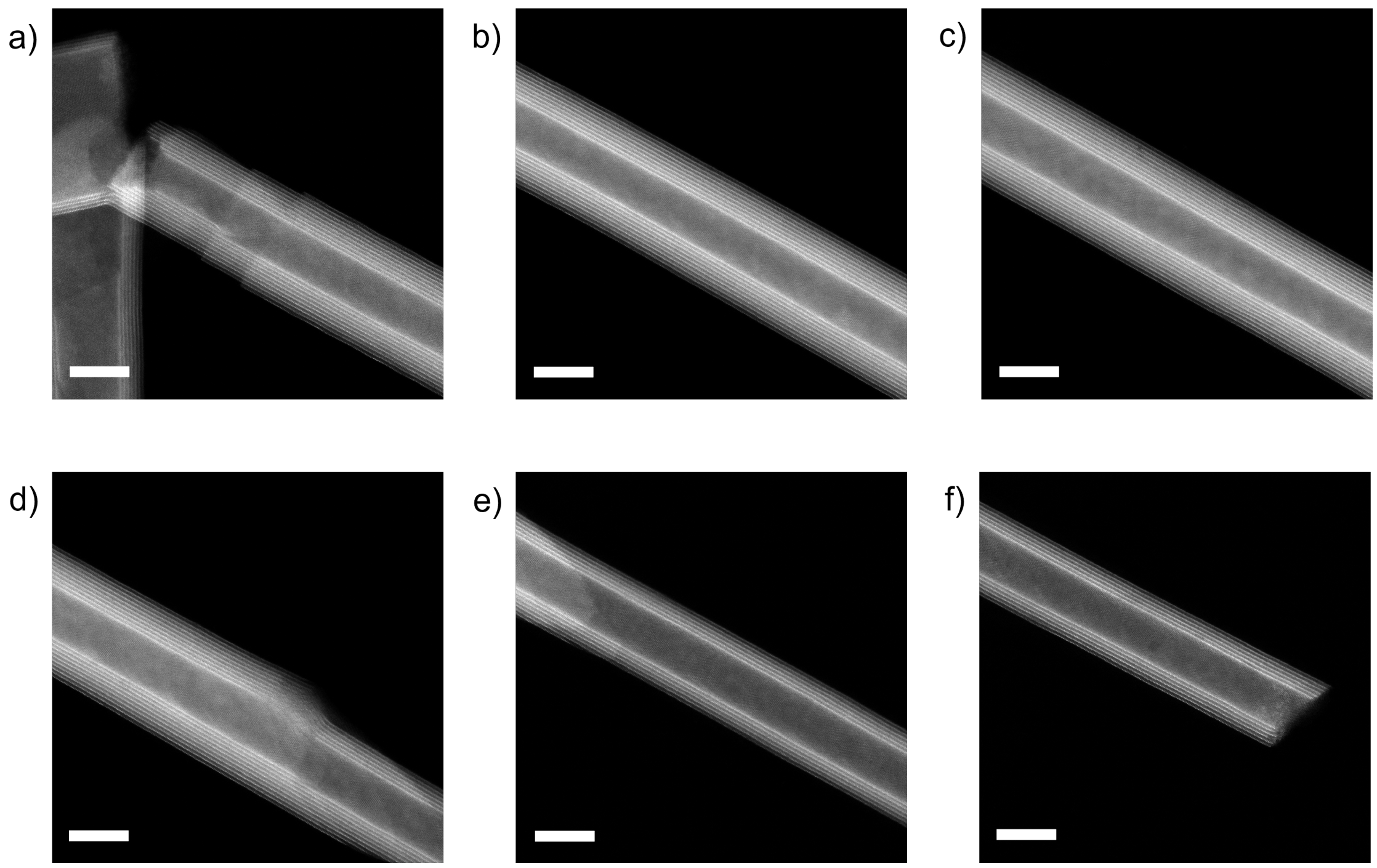

Figure S9: **Additional HRSTEM images of hollow WS_2 nanotubes grown from 5 nm W films.** **(a)** Root-proximal region showing multilayer WS_2 shells around a hollow interior. **(b, c)** Nanotube body regions where the WS_2 shells remain comparatively uniform and concentric. **(d, e)** Segments showing local variation in wall thickness and partial interruption of the inner shells. **(f)** Open or tapered nanotube end with a reduced number of shells. These images show that the 5 nm-derived nanotubes commonly contain hollow interiors and regular concentric WS_2 walls, while local shell interruptions remain spatially limited. Scale bars: 10 nm.

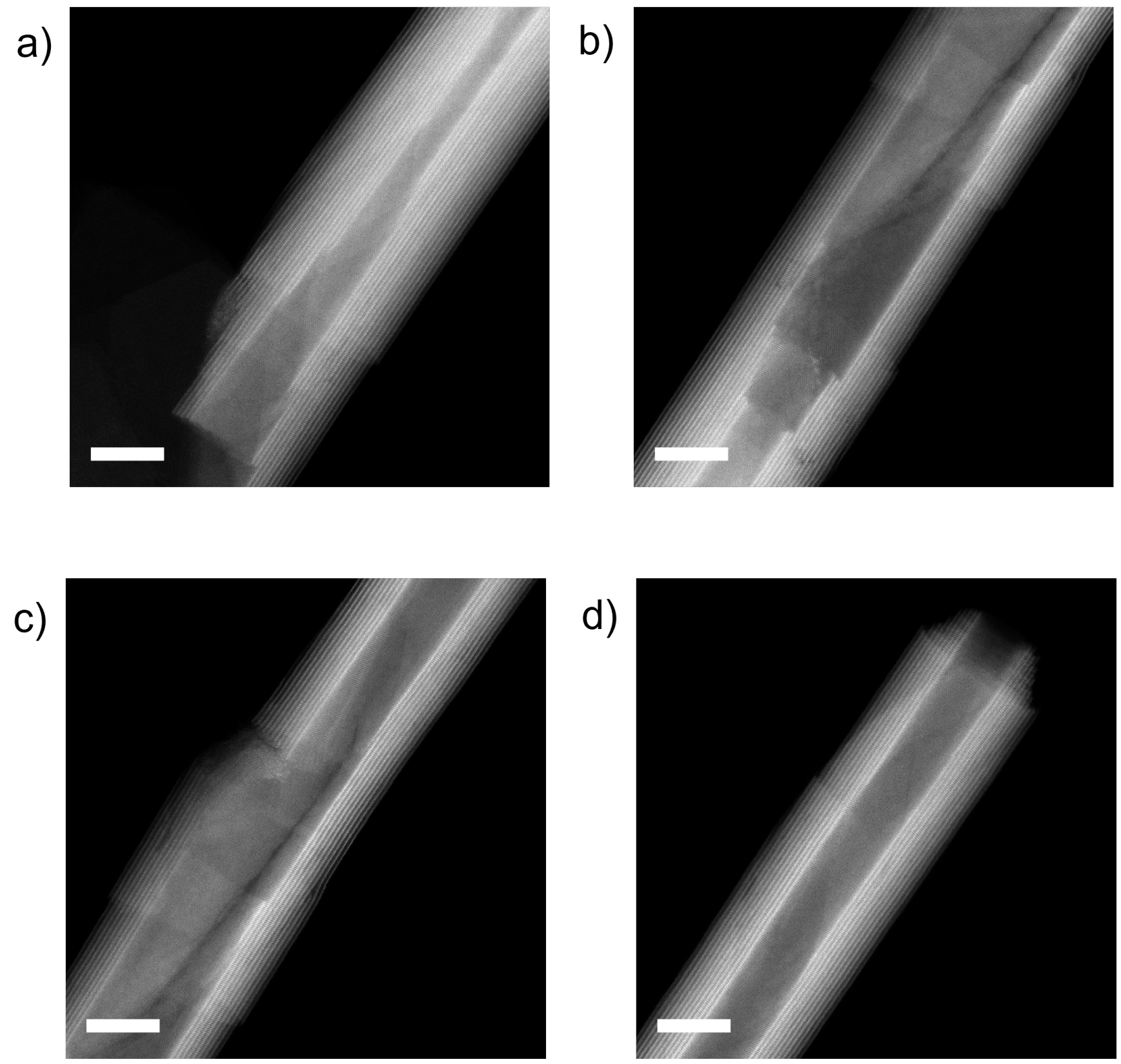

Figure S10: **Local shell interruption and recovery in a 5 nm-derived WS_2 nanotube.** **(a)** Nanotube segment close to the root, showing concentric WS_2 shells around a hollow interior. **(b)** Region with a local interruption of the inner shell continuity and variation in wall thickness. **(c)** Segment after the interrupted region, where the concentric shell arrangement is recovered. **(d)** Open nanotube end showing comparatively symmetric shell structure. These images indicate that local defects or shell interruptions can occur in 5 nm-derived nanotubes, but they do not dominate the overall hollow concentric shell morphology. Scale bars: 10 nm.

S8 Additional HRSTEM analysis of 10 nm W/Si nanotubes

The 10 nm W/Si sample represents an intermediate conversion regime between the hollow, island-associated nanotubes obtained from 5 nm W films and the retained-core nanotubes obtained from 100 nm W films. The main text shows that a representative 10 nm-derived nanotube contains root-proximal regions with comparatively symmetric WS_2 shells, a mid-

dle region with a high-contrast axial remnant, and a tip-proximal region with local shell wrapping. The additional HRSTEM and FFT analyses in this section provide supporting evidence for this axially heterogeneous conversion pathway.

Figure S11 shows a second representative 10 nm-derived nanotube with a hollow wall structure and local shell-number asymmetry. The intensity profiles extracted across the boxed regions show high-contrast WS_2 wall fringes separated by a lower-intensity hollow core. The profiles also show that the apparent shell number can differ between opposite tube walls, consistent with local shell asymmetry during conversion.

Figure S12 provides local FFT analysis of remnant-rich and shell-rich regions in a 10 nm-derived nanotube. The WS_2 shell region shows a spacing close to the expected interlayer distance for layered WS_2, whereas the remnant-rich regions show additional periodicities distinct from the dominant WS_2 shell spacing. These additional periodicities are consistent with a locally retained WO_x-derived remnant, but the overlap among tungsten oxide and suboxide lattice spacings prevents assignment of a unique oxide phase from FFT analysis alone. Together, Figs. S11–S12 support the main-text interpretation that 10 nm-derived nanotubes undergo axially heterogeneous oxide-template conversion with local shell asymmetry and incomplete oxide-core recession.

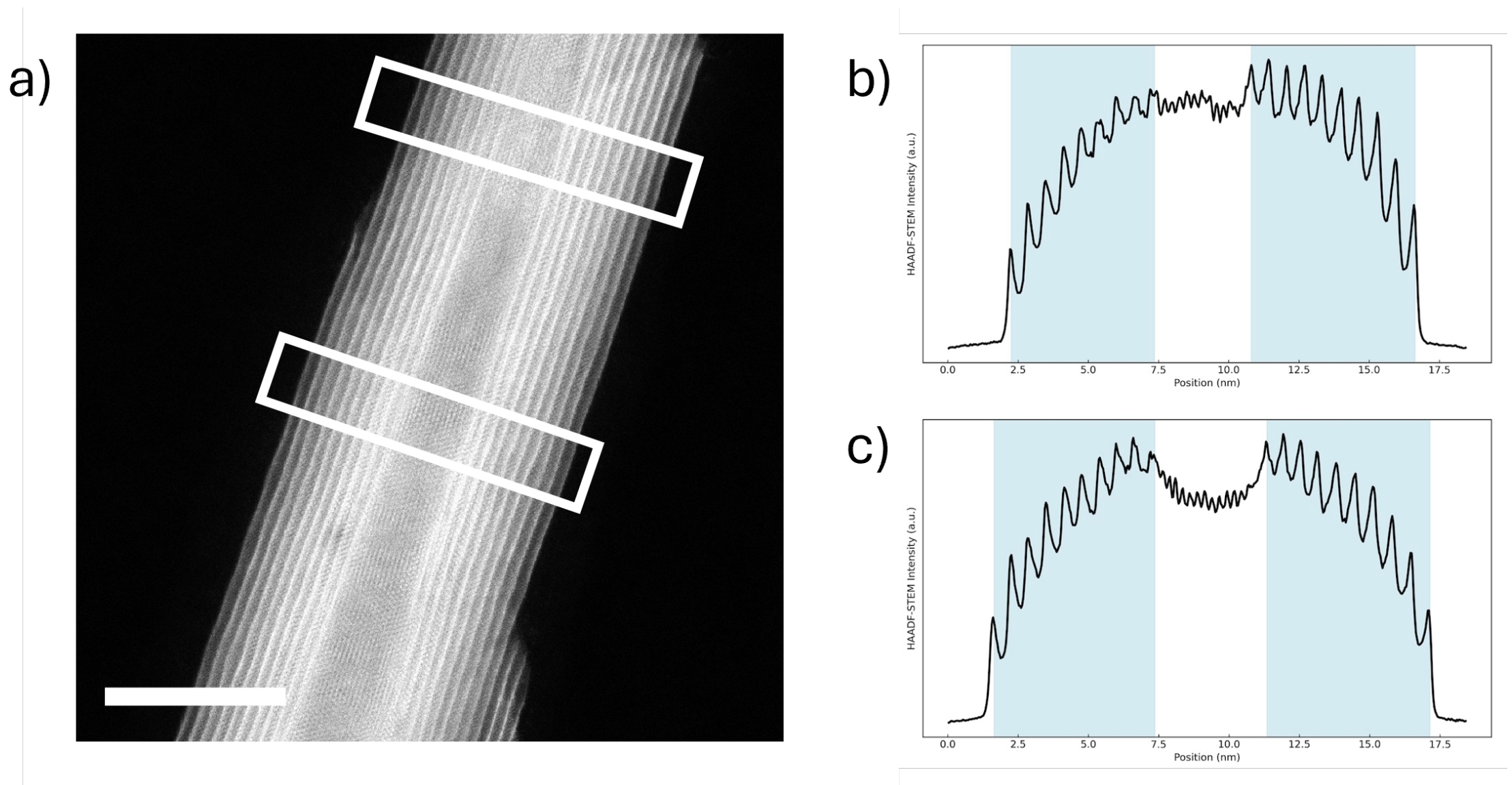

Figure S11: **Asymmetric and symmetric wall structures in a WS_2 nanotube grown from a 10 nm W film.** **(a)** HAADF-STEM image of a second representative nanotube showing a hollow WS_2 wall structure with local shell asymmetry; white boxes indicate the regions used for line-profile analysis. Scale bar: 10 nm. **(b,c)** Intensity profiles extracted from the boxed regions in **(a)**, showing high-contrast WS_2 wall fringes separated by a lower-intensity hollow core. The profiles reveal local variation in shell number between opposite tube walls.

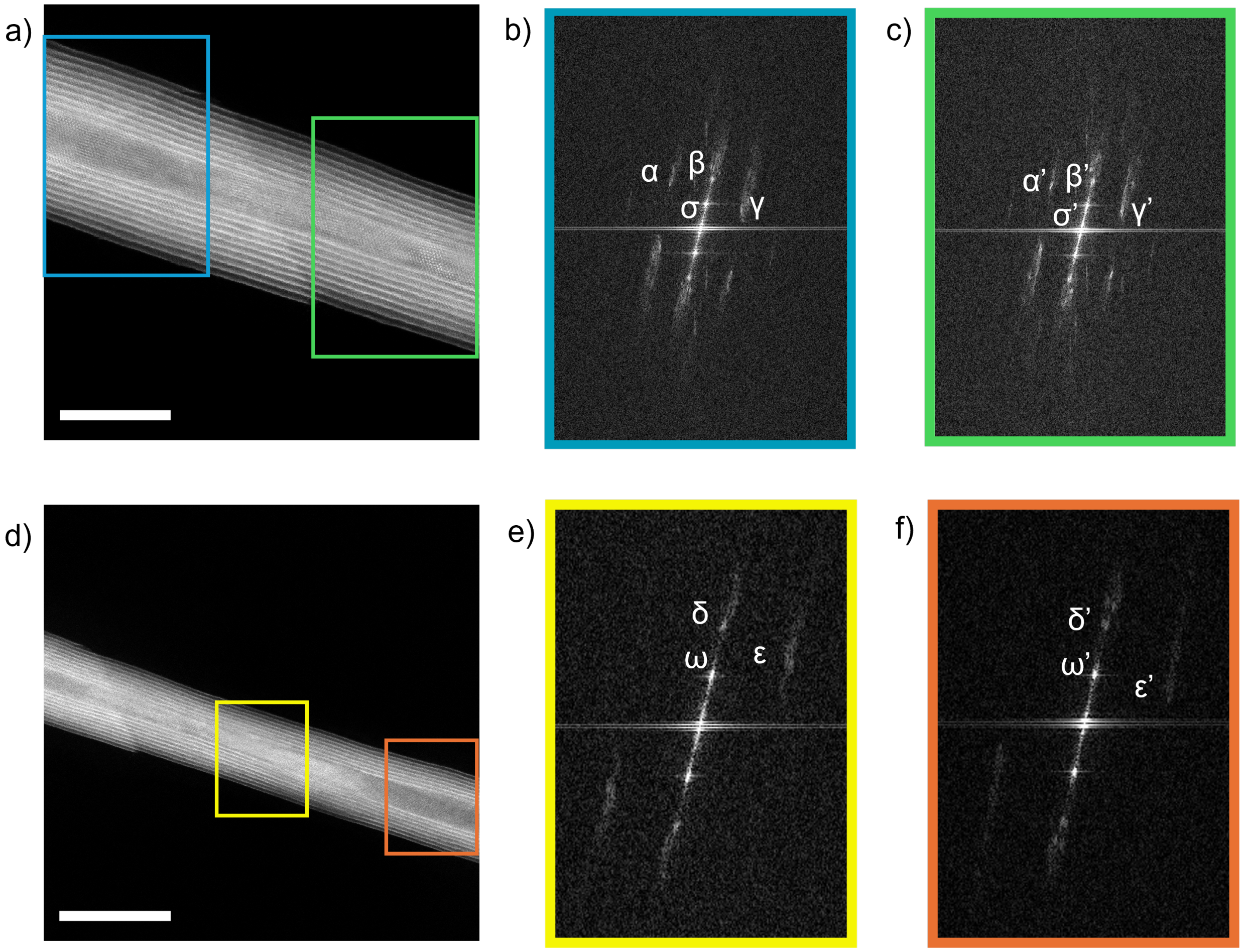

Figure S12: **Local FFT analysis of a 10 nm-derived WS_2 nanotube containing a WO_x-derived remnant.** **(a)** HAADF-STEM image of a nanotube region near the tip-proximal segment shown in Fig. 4, with two regions of interest selected for FFT analysis. The blue box corresponds to a region closer to the hollow core, and the green box corresponds to a region closer to the high-contrast remnant. **(b,c)** FFTs extracted from the blue and green regions in **(a)**, showing additional lattice periodicities distinct from the dominant WS_2 shell spacing. **(d)** HAADF-STEM image of another nanotube region, with the yellow box highlighting the high-contrast remnant and the orange box highlighting the WS_2 shell region. **(e,f)** FFTs extracted from the boxed regions in **(d)**. The remnant-rich region shows a spacing of approximately 0.23 nm, while the WS_2 shell region shows a spacing of approximately 0.60 nm, consistent with layered WS_2. The additional periodicities are consistent with a locally retained WO_x-derived remnant, but the overlap among tungsten oxide and suboxide lattice spacings prevents assignment of a unique oxide phase from FFT analysis alone. Scale bars: **(a)** 10 nm and **(d)** 20 nm.

Table S6: Measured d-spacings and inter-spot angles obtained from the FFT of 10 nm-derived WS_2 nanotubes. Measured interplanar spacings d as well as measured angles between reflections. Within each group of reflections (α–σ, α'–σ', δ–ω, δ'–ω'), the first reflection (α, α', δ and δ', respectively) is set as the angular reference at $\theta = 0°$, and the angles quoted for the remaining reflections are measured with respect to this reference reflection.

Diffraction Spot	Interplanar Spacing d [nm]	θ [°]
α	0.2562	0
β	0.3090	47.73
γ	0.2637	100.23
σ	0.6182	47.14
α'	0.2638	0
β'	0.3188	47.44
γ'	0.2628	98.82
σ'	0.6576	48.49
δ	0.3161	0
ϵ	0.2266	1.32
ω	0.6323	39.86
δ'	0.3211	0
ϵ'	0.2625	0.92
ω'	0.6468	58.25

S9 HRSTEM analysis of 100 nm W/Si nanotubes

S9.1 Retained-core morphology and nonuniform shell development

HRSTEM images of nanotubes grown from 100 nm W films show retained-core and nonuniform shell morphologies consistent with the thick-film conversion pathway discussed in the main text. In these nanotubes, multilayer WS_2 shells surround regions of enhanced HAADF contrast assigned to retained WO_x-derived material. The shell thickness, shell registry, and retained-core contrast vary along the nanotube axis, indicating that sulfurization and oxide-core recession do not proceed uniformly in the 100 nm regime.

Figures S13 and S14 show representative 100 nm-derived nanotubes with retained-core contrast and nonuniform WS_2 shell development. These images support the interpretation that the large precursor volume in 100 nm W films prevents uniform hollow conversion. They

do not, however, require assignment to a unique dual-front sulfurization mechanism.

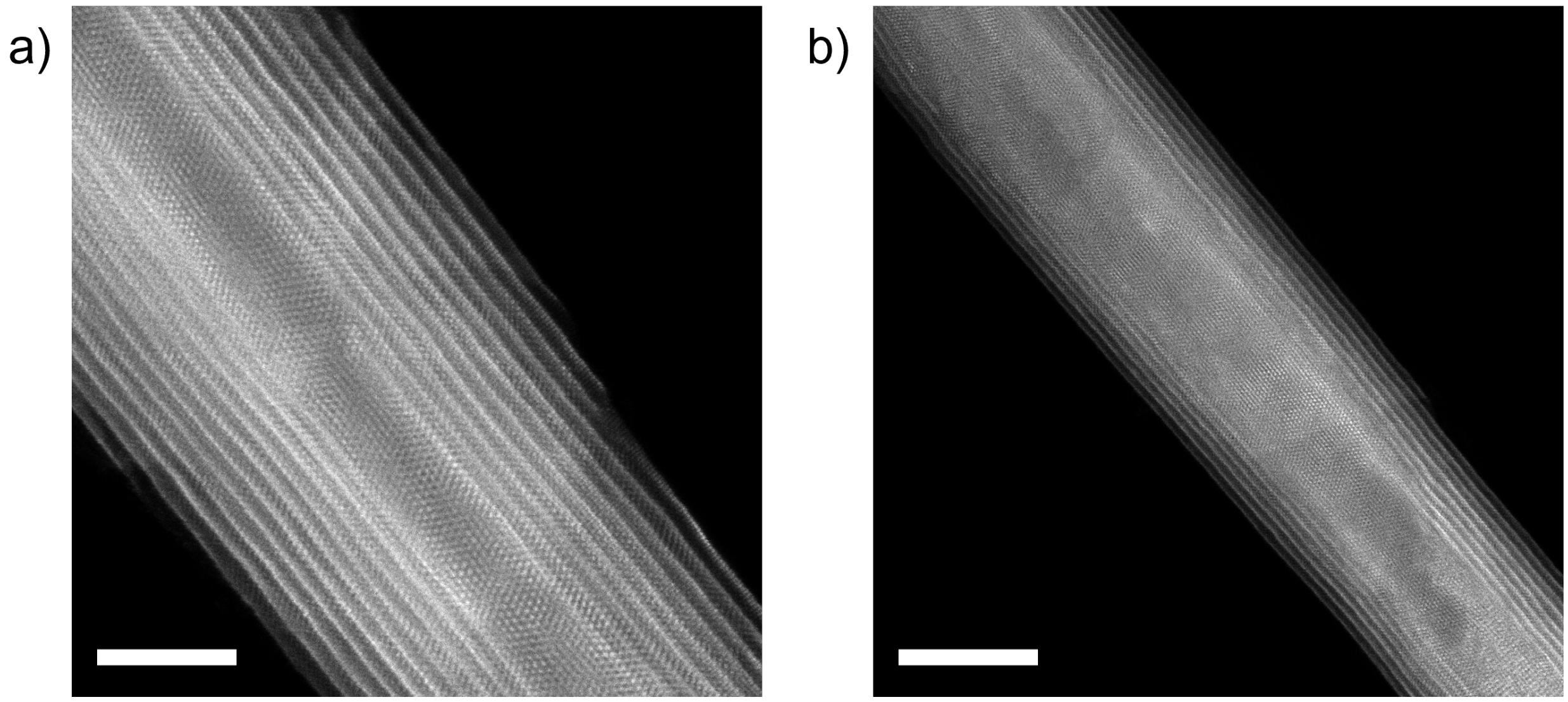

Figure S13: **Retained-core and nonuniform shell morphology in a WS_2 nanotube derived from a 100 nm W film.** **(a)** High-magnification HRSTEM image of an upper-region nanotube segment showing multilayer WS_2 shells and local shell-front nonuniformity. Scale bar: 5 nm. **(b)** Lower-magnification HRSTEM image of the same nanotube segment, showing nonuniform shell development along the nanotube axis. Scale bar: 10 nm. These images provide an additional example of retained-core conversion in the 100 nm regime.

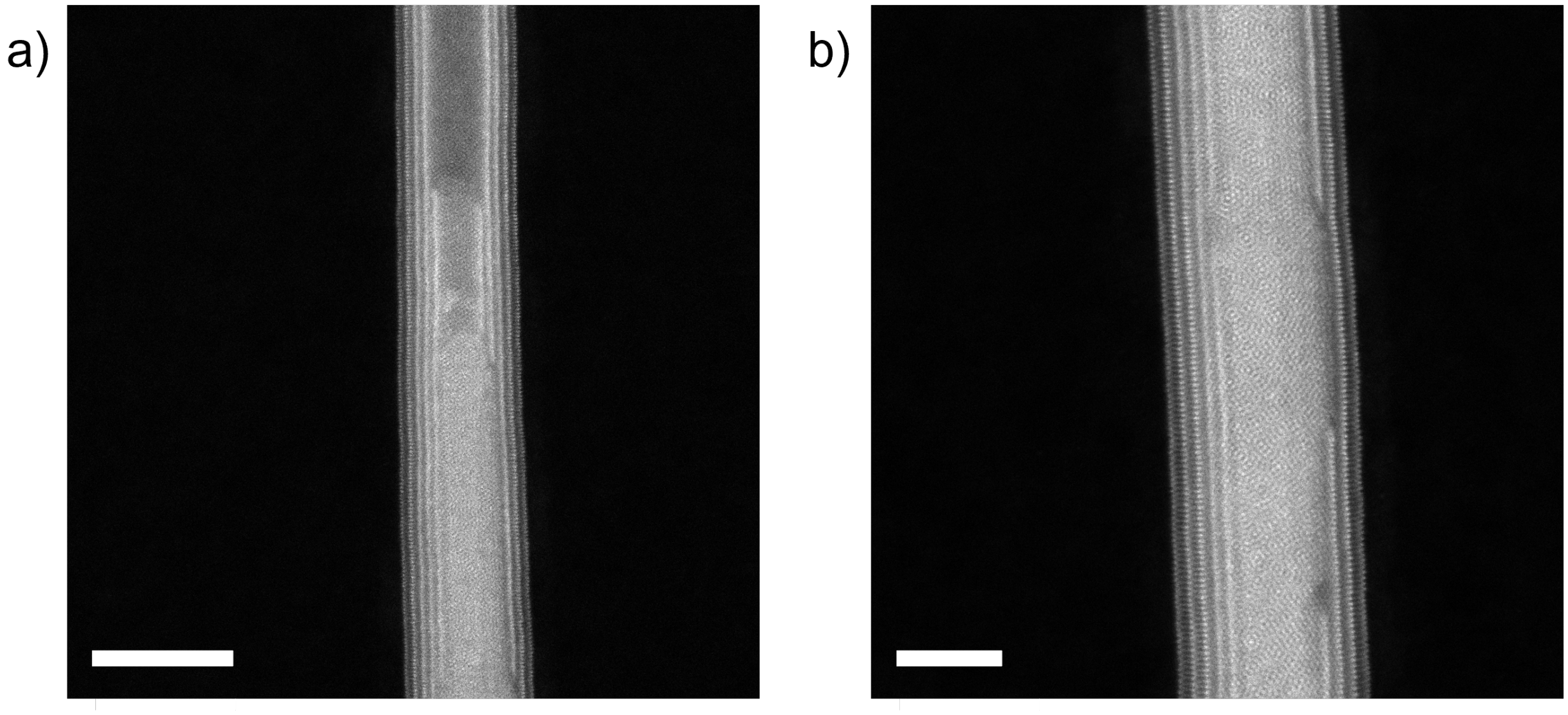

Figure S14: **Retained-core WS_2 nanotube morphology derived from a 100 nm W film.** **(a)** HRSTEM image showing a multilayer WS_2 shell surrounding a retained WO_x-derived core region. Scale bar: 10 nm. **(b)** Higher-magnification HRSTEM image showing the interface between the retained WO_x-derived core and the surrounding WS_2 shell. Scale bar: 5 nm. The images illustrate incomplete oxide-core recession and nonuniform shell development in the thick-film regime.

S9.2 Shell-front mismatch and spiral-like secondary wrapping

Some 100 nm-derived nanotubes show local spiral-like WS_2 shell wrapping at regions where shell fronts or retained-core geometries become nonuniform (Fig. S15). In one example, an external spiral-like layer is observed near a local shell-front discontinuity (Fig. S15a). In another example, asymmetric recession of the retained WO_x-derived core is associated with internal spiral-like shell wrapping (Fig. S15b). These observations support the interpretation that nonuniform conversion in the thick-film regime can generate persistent step-like shell edges associated with secondary wrapping.

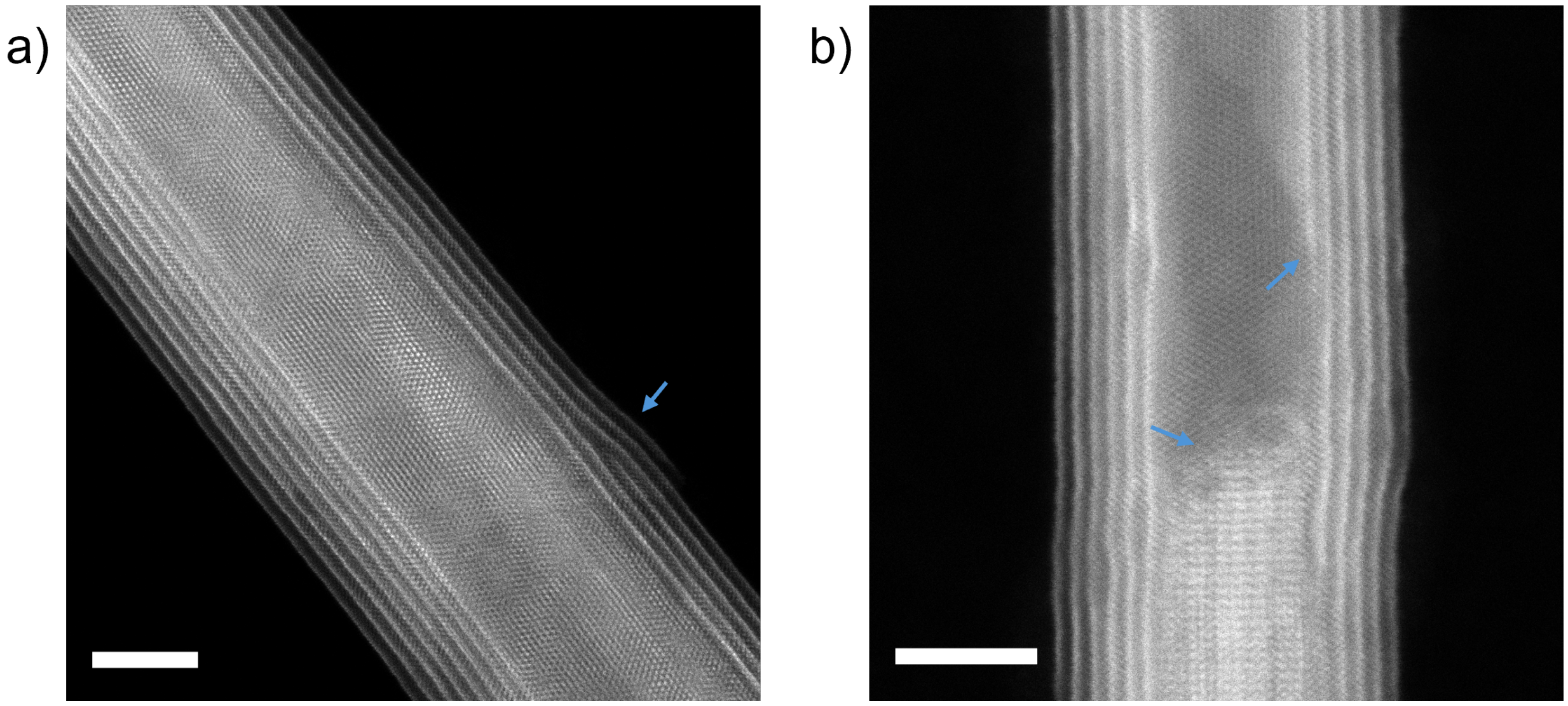

Figure S15: **Spiral-like shell wrapping in 100 nm-derived WS_2 nanotubes. (a)** HRSTEM image of a multiwall WS_2 nanotube showing an external spiral-like layer near a local shell-front discontinuity, highlighted by the blue arrow. Scale bar: 5 nm. **(b)** HRSTEM image of a retained-core WO_x-derived/WS_2-shell nanotube showing asymmetric retained-core recession and internal spiral-like shell wrapping, highlighted by the blue arrows. Scale bar: 5 nm. These images support the interpretation that nonuniform conversion in the 100 nm regime can generate step-like shell edges associated with secondary shell wrapping.

Line-profile analysis across one shell-front mismatch region shows interlayer spacings close to 0.623 nm on both sides of the discontinuity (Fig. S16). The similar spacings indicate that the feature is better described as local shell misregistration within WS_2, rather than gradual elastic distortion across the tube wall. Together, Figs. S15–S16 support the main-text interpretation that retained-core conversion in the 100 nm regime can produce local shell-front mismatch and spiral-like secondary shell wrapping, without requiring assignment to a resolved axial screw dislocation.

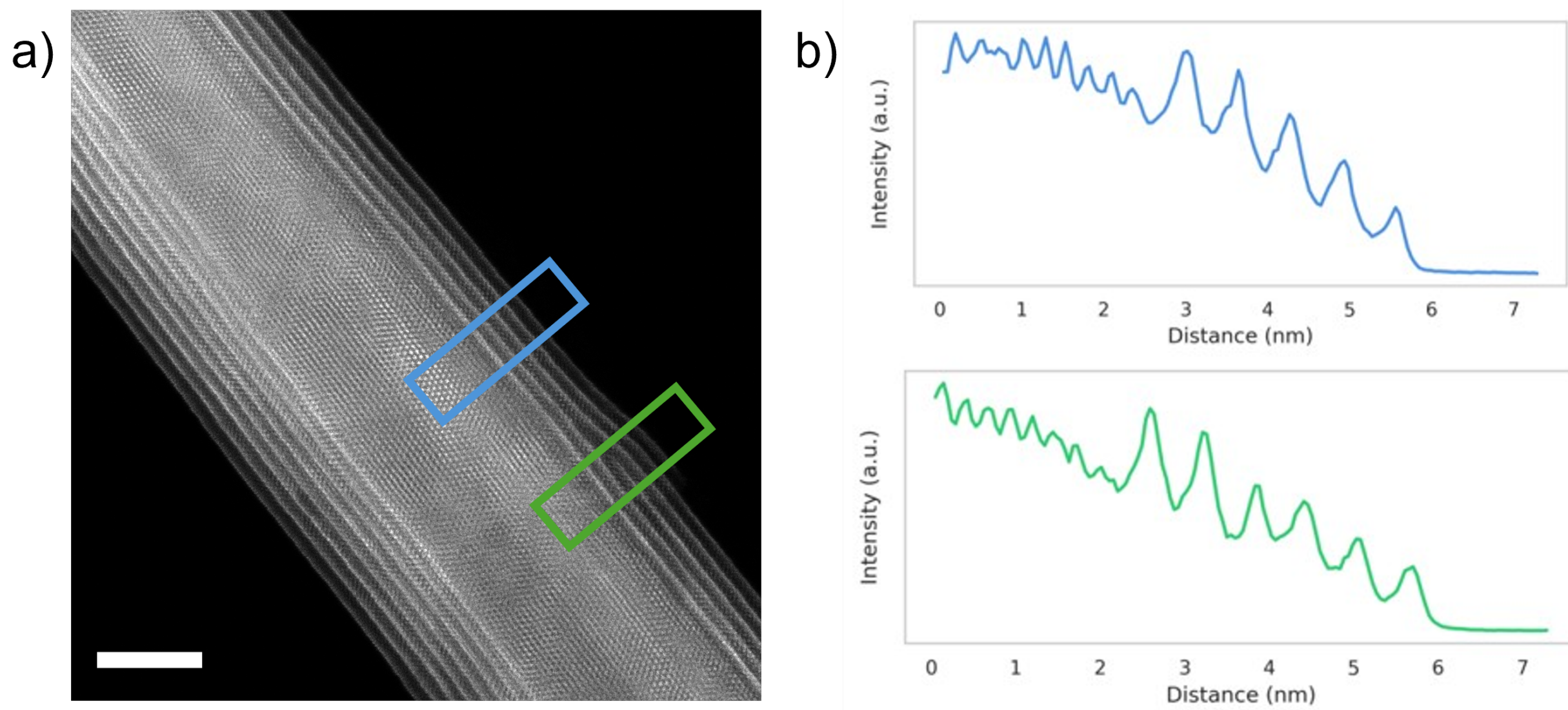

Figure S16: **Structural and intensity analysis of a shell-front mismatch region in a partially sulfurized WS_2 nanostructure formed from a 100 nm W film. (a)** HAADF-STEM image showing a nanotube segment containing a local shell-front discontinuity. The boxed regions indicate where intensity profiles were extracted: blue corresponds to a region away from the discontinuity, and green corresponds to a region crossing the discontinuity. Scale bar: 5 nm. **(b)** Line profile from the blue region showing an interlayer spacing of approximately 0.623 nm. **(c)** Line profile from the green region also showing interlayer distances close to 0.623 nm. The similar spacings support local shell misregistration within WS_2, rather than gradual elastic distortion across the tube wall.

S10 Nanotube length and diameter statistics

Nanotube length and diameter statistics were obtained from WS_2 nanotubes transferred from the growth substrate onto TEM grids. Nanotubes derived from 100, 10, and 5 nm W/Si samples were analyzed, with $N = 28$ nanotubes measured for each initial W film thickness. Visibly broken, damaged, or strongly bent nanotubes were excluded from the analysis. Because mechanical transfer to TEM grids can affect the measured length distribution, and because the sample size is limited, these values are used to support relative morphology trends rather than to quantify absolute substrate-scale growth yields.

For nanotubes derived from 100 nm W/Si, the length distribution is broad, with a mean length of 1.521 μm and a standard deviation of 0.733 μm (Fig. S17a). The corresponding diameter distribution gives a mean diameter of 48.58 nm and a standard deviation of 15.58 nm

(Fig. S17b). The ratio of the mean length to the mean diameter is approximately 31. This large mean diameter and broad diameter distribution are consistent with the retained-core and spatially nonuniform conversion morphology observed for the 100 nm-derived nanotubes in the main text.

For nanotubes derived from 10 nm W/Si, the mean length is 0.623 μm with a standard deviation of 0.211 μm (Fig. S17c), while the mean diameter is 26.85 nm with a standard deviation of 8.37 nm (Fig. S17d). The ratio of the mean length to the mean diameter is approximately 23. These values show that the 10 nm-derived nanotubes are shorter and have smaller diameters than the 100 nm-derived nanotubes, while still forming a dense nanotube-rich morphology.

For nanotubes derived from 5 nm W/Si, the mean length is 0.514 μm with a standard deviation of 0.251 μm (Fig. S17e), and the mean diameter is 28.51 nm with a standard deviation of 8.58 nm (Fig. S17f). The ratio of the mean length to the mean diameter is approximately 18. Compared with the 10 nm-derived nanotubes, the 5 nm-derived nanotubes have a similar mean diameter within the measured spread but a shorter mean length, consistent with the sparse, island-associated growth morphology observed for the 5 nm regime.

Overall, the 100 nm-derived nanotubes show the largest mean diameter and the broadest diameter distribution, whereas the 10 and 5 nm-derived nanotubes have smaller and broadly comparable diameters. The length distributions decrease from the 100 nm regime to the 10 and 5 nm regimes, consistent with the transition from dense thick-film-derived nanotube networks to shorter island-associated nanotubes. These trends support a thickness-dependent change in nanotube dimensions and morphology, rather than a simple monotonic decrease in all nanotube dimensions with decreasing W film thickness.

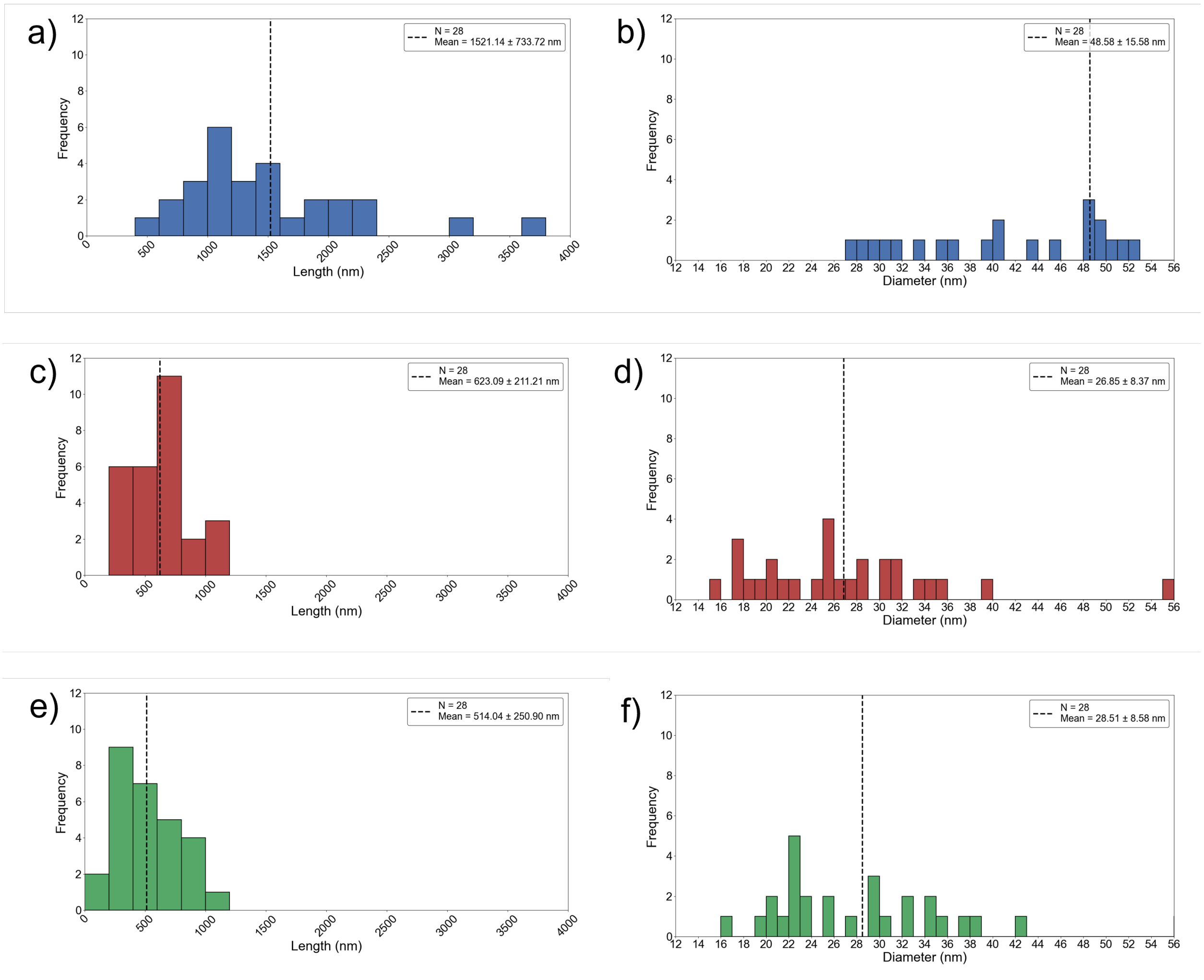

Figure S17: **Nanotube length and diameter statistics for different initial W film thicknesses.** Measurements were performed on nanotubes transferred onto TEM grids, with $N = 28$ nanotubes analyzed for each initial W film thickness. Visibly broken, damaged, or strongly bent nanotubes were excluded from the analysis. **(a,b)** Length and diameter distributions for nanotubes derived from 100 nm W/Si. **(c,d)** Length and diameter distributions for nanotubes derived from 10 nm W/Si. **(e,f)** Length and diameter distributions for nanotubes derived from 5 nm W/Si. The corresponding mean values and standard deviations are summarized in Section S10.

S11 Multiphoton microscopy of WS_2 nanotubes

Figure S18 illustrates a sketch of the experimental setup used to investigate the nonlinear response of WS_2-based samples.[3,4] A *Light Conversion Pharos* femtosecond laser oscillator (PH1-10-0400-02-10-sP) and a *Light Conversion Orpheus* tunable optical parametric oscil-

lator (OPA) deliver two linearly polarized beams at λ_1 and λ_2, respectively, with pulse durations on the order of $\Delta\tau =$ 200 fs. The first pulse, fixed at $\lambda_1 = 1030$ nm, passes through a long optical delay line to compensate for the additional optical path traveled by the second pulse inside the OPO system. Additionally, the delay time between the pulses is controlled using a retro reflector cube and a motorized optical delay line (Thorlabs LTS300C/M) that sets the optical path of the first pulse. The second pulse can be tuned in the range $\lambda_2 =$ 750-850 nm. Both pulses pass through energy control systems that consist of two separate motorized super-achromatic half-wave plates ($\lambda/2$) and high-power Glan-Taylor polarizers (PBC). By employing a dichroic mirror both laser pulses are recombined along the same optical path and steered toward an infinity corrected microscope objective (*Olympus* UPLSAPO 40× objective, NA = 0.95) that focuses them on the sample. The sample position can be controlled using a motorized xyz-stage. After laser excitation, the resultant nonlinear signal generated at the sample is collected by the objective, steered by a second dichroic mirror, filtered (Thorlabs FESH700) and directed towards a spectrometer (Ocean Insight QEPro). In addition, we used an in-situ optical microscope to inspect the surface of the sample during the experiments. Incoherent light produced by an LED is coupled into the beam path by a pellicle beam splitter. The portion of the light reflected by the surface of the sample is steered towards a CMOS camera (Thorlabs Zelux) using the same path as the spectrometer system.

Power-normalized FWM enhancement estimate

For the FWM comparison in Fig. 6d, the power-normalized detected enhancement is estimated as

$$\eta_{\mathrm{FWM}}^{\mathrm{det}} = \frac{S_{\mathrm{NT}}}{S_{\mathrm{ML}}} \left(\frac{P_{\lambda_2,\mathrm{ML}}}{P_{\lambda_2,\mathrm{NT}}} \right)^2 = \frac{50}{1.9} \left(\frac{400}{20} \right)^2 \approx 1.05 \times 10^4, \tag{S1}$$

where S_{NT} and S_{ML} are the raw detected FWM peak count rates and $P_{\lambda_2,\mathrm{NT}}$, $P_{\lambda_2,\mathrm{ML}}$ the corresponding tunable-beam powers. Taking the detected FWM intensity to scale as $|\chi^{(3)}|^2$,

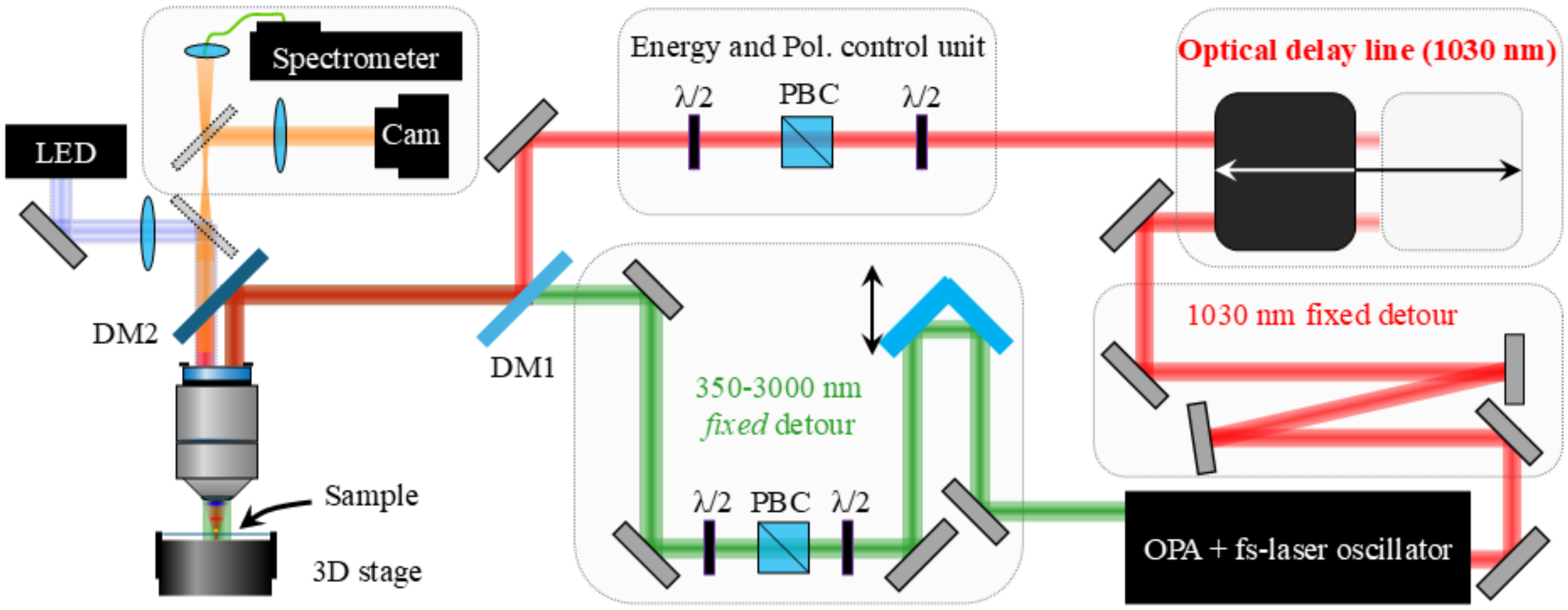

Figure S18: Setup for characterization of the nonlinear signal emitted by WS_2 nanotubes upon femtosecond multiphoton laser illumination

this corresponds to an apparent field-level ratio

$$\frac{|\chi^{(3)}_{\mathrm{NT}}|}{|\chi^{(3)}_{\mathrm{ML}}|} \approx \left(\eta^{\mathrm{det}}_{\mathrm{FWM}}\right)^{1/2} \approx 1.0 \times 10^2. \tag{S2}$$

As discussed in the main text, this value is an effective detected contrast, not an intrinsic $\chi^{(3)}$ ratio, because the nanotube assemblies and the monolayer differ in illuminated volume, wall number, orientation, local environment and collection efficiency.

References

(1) Diaz-Coello, S.; Winkler, D.; Griesser, C.; Moser, T.; Rodríguez, J. L.; Kunze-Liebhäuser, J.; García, G.; Pastor, E. Highly active W_2C-based composites for the HER in alkaline solution: the role of surface oxide species. *ACS Applied Materials & Interfaces* **2024**, *16*, 21877–21884.

(2) Wang, B.; Zhong, X.; Xu, H.; Zhang, Y.; Cvelbar, U.; Ostrikov, K. Structure and photoluminescence of WO_{3-x} aggregates tuned by surfactants. *Micromachines* **2022**, *13*, 2075.

(3) Hernandez-Rueda, J.; Noordam, M. L.; Komen, I.; Kuipers, L. Nonlinear Optical Response of a WS_2 Monolayer at Room Temperature upon Multicolor Laser Excitation. *ACS Photonics* **2021**, *8*, 550–556.

(4) Vreugdenhil, M.; Hernandez-Rueda, J.; van Oosten, D. Wavelength Dependence of the Femtosecond Laser Ablation Threshold in Band-Gap Materials. **2026**, Preprint, available at SSRN: https://ssrn.com/abstract=6748673.
